# Matrix association region/scaffold attachment region: the crucial player in defining the positions of chromosome breaks mediated by bile acid-induced apoptosis in nasopharyngeal epithelial cells

**DOI:** 10.1186/s12920-018-0465-4

**Published:** 2019-01-15

**Authors:** Sang-Nee Tan, Sai-Peng Sim

**Affiliations:** Faculty of Medicine and Health Sciences, Department of Paraclinical Sciences, Universiti Malaysia Sarawak, Kota Samarahan, Sarawak Malaysia

**Keywords:** Chronic rhinosinusitis, Nasopharyngeal carcinoma, Gastro-oesophageal reflux, Bile acid, Apoptosis, AF9, Matrix association region/scaffold attachment region

## Abstract

**Background:**

It has been found that chronic rhinosinusitis (CRS) increases the risk of developing nasopharyngeal carcinoma (NPC). CRS can be caused by gastro-oesophageal reflux (GOR) that may reach nasopharynx. The major component of refluxate, bile acid (BA) has been found to be carcinogenic and genotoxic. BA-induced apoptosis has been associated with various cancers. We have previously demonstrated that BA induced apoptosis and gene cleavages in nasopharyngeal epithelial cells. Chromosomal cleavage occurs at the early stage of both apoptosis and chromosome rearrangement. It was suggested that chromosome breaks tend to cluster in the region containing matrix association region/scaffold attachment region (MAR/SAR). This study hypothesised that BA may cause chromosome breaks at MAR/SAR leading to chromosome aberrations in NPC. This study targeted the *AF9* gene located at 9p22 because 9p22 is a deletion hotspot in NPC.

**Methods:**

Potential MAR/SAR sites were predicted in the *AF9* gene by using MAR/SAR prediction tools. Normal nasopharyngeal epithelial cells (NP69) and NPC cells (TWO4) were treated with BA at neutral and acidic pH. Inverse-PCR (IPCR) was used to identify chromosome breaks in SAR region (contains MAR/SAR) and non-SAR region (does not contain MAR/SAR). To map the chromosomal breakpoints within the *AF9* SAR and non-SAR regions, DNA sequencing was performed.

**Results:**

In the *AF9* SAR region, the gene cleavage frequencies of BA-treated NP69 and TWO4 cells were significantly higher than those of untreated control. As for the *AF9* non-SAR region, no significant difference in cleavage frequency was detected between untreated and BA-treated cells. A few breakpoints detected in the SAR region were mapped within the *AF9* region that was previously reported to translocate with the mixed lineage leukaemia (*MLL*) gene in an acute lymphoblastic leukaemia (ALL) patient.

**Conclusions:**

Our findings suggest that MAR/SAR may be involved in defining the positions of chromosomal breakages induced by BA. Our report here, for the first time, unravelled the relation of these BA-induced chromosomal breakages to the *AF9* chromatin structure.

**Electronic supplementary material:**

The online version of this article (10.1186/s12920-018-0465-4) contains supplementary material, which is available to authorized users.

## Background

Genetic alterations [1], epigenetic changes [2], and environmental factors [3] are thought to be involved in the development of nasopharyngeal carcinoma (NPC). Several environmental risk factors that contribute to NPC have been identified. These include Epstein-Barr virus (EBV) infection [4, 5], dietary exposure to nitrosamines [6] as well as occupational exposure to smokes, wood dust, formaldehyde and intense industrial heat [6–8]. In addition, prior history of chronic nose and ear diseases (such as chronic rhinitis, sinusitis and otitis media) has also long been recognised as a risk factor for developing NPC [9–15]. Individuals with chronic rhinosinusitis (CRS), the inflammation of the nose and paranasal sinuses, have been shown to have a significantly higher risk of developing NPC as compared with the control individuals without CRS [15]. Although chronic inflammation of nose or ear has long been recognised as a risk factor for NPC, the underlying mechanisms by which this risk factor may contribute to NPC pathogenesis remain elusive.

Gastro-oesophageal reflux disease (GORD) is one of the major aetiological factors of chronic inflammation of sinonasal tract or ear [16–20]. GORD is caused by the flowing back of gastric duodenal contents into the oesophagus. It has been reported that the gastric duodenal refluxate may flow beyond the oesophagus. In turn, the gastric duodenal contents may affect the tracheobronchopulmonary tree, larynx, pharynx, sinonasal tract and middle ear [18, 21, 22]. The typical GORD symptoms such as heartburn and acid regurgitation may not be present in half of these patients [19]. Thus, these atypical manifestations of GORD are not only referred as extraoesophageal reflux (EOR) or laryngopharyngeal reflux [18, 23] but also as ‘silent reflux’ [19].

GORD is related to various inflammatory disorders. These inflammatory disorders include gastritis [24, 25], oesophagitis [26–28], laryngitis [29–31], pharyngitis [32, 33], post-nasal drip [34], otitis media [35–38] and asthma [39–41]. Moreover, the relation between CRS and GORD has increasingly received much concern [33, 42, 43]. It has been reported that individual with GORD has a significantly higher risk of developing CRS [44]. The prevalence of acid pharyngeal reflux in patients with CRS has been found to be higher than that in the normal controls (64% vs 18%) [42]. Seventy-eight percent of patients with CRS has been observed to have GOR [45]. Nasopharyngeal reflux has been demonstrated in both paediatric [46–49] and adult groups [34, 42, 43, 50].

Besides, GORD has also been related to various cancers. These cancers include stomach cancer [51, 52], oesophageal adenocarcinoma [53, 54], laryngeal cancer [55], pharyngeal cancer [56] and lung cancer [57]. Bile acid (BA), the major component of acid refluxate has been identified as a carcinogen in human malignancies (reviewed in [58]). It has been found that the levels of total pepsin and BA in the saliva of patients with laryngopharyngeal reflux were approximately three-fold higher than those of the normal volunteers [59]. It has also been reported that the levels of total pepsin and BA in the saliva of early laryngeal carcinoma patients were about four-fold higher than those of the normal controls [60]. In addition, BA has also been shown to have the carcinogenic effect in human hypopharyngeal squamous carcinoma FaDu cells through epithelial-mesenchymal transition (EMT) [61]. EMT is a major pathway related to cancer invasion and metastasis [62]. These observations suggested a potential role for biliary reflux in the pathogenesis of laryngeal and pharyngeal cancers.

There are strong associations among oxidative stress, inflammation and cancer [63–65]. Oxidative stress may activate nuclear factor-kappa B (NF-kappa B) [66] which plays a vital role in inflammatory response [67]. The activation of this transcription factor leads to the expression of genes involved in inflammation [66]. On the other hand, the inflammatory condition generates excessive reactive oxygen species (ROS) in cells. The free radicals may interact directly with DNA or interfere with DNA repair system. These, in turn, elevate the mutation rate in the inflammatory cells. Therefore, chronic inflammation predisposes the cells to neoplastic transformation. Cytokines have been found to be the important mediators that relate inflammation to cancer through oxidative stress [68]. It has been demonstrated that the combination of BA and acid triggered NF-kappa B activation in human hypopharyngeal epithelial cells. This, in turn, leads to overexpression of genes associated with antiapoptosis and oncogenic properties [69]. NF-kappa B pathway is well known to be a proinflammatory signalling pathway. This pathway is mainly activated by proinflammatory cytokines such as interleukin 1 (IL-1) and tumour necrosis factor-alpha (TNF-alpha) [70]. ROS are known to act as the messengers in NF-kappa B activation. It has been found that the anti-inflammatory cytokine IL-10 was able to inhibit the NF-kappa B activation in the stimulated macrophages via reduction of ROS [71].

It has recently been reported that the level of BA in the serum of NPC patients was significantly higher than that of the normal controls. The level of BA in the serum of NPC patients significantly inhibited the secretion of the IL-10 protein in CD4+ CD5- T cells [72]. IL-10 is suggested to have an anti-inflammatory role through reduction of oxidative stress induced by the proinflammatory factors. Treatment of Caco-2 intestinal epithelial cells with proinflammatory factors such as TNF-alpha, serotonin, adenosine and melatonin has been shown to induce oxidative damage in proteins and lipids. IL-10 was found to be able to reverse the oxidative damage by restoring the activities of antioxidant enzymes such as catalase, superoxide dismutase and glutathione peroxidise [73]. It has also been demonstrated that IL-10 inhibited hydrogen peroxide (H_2_O_2_) generation triggered by interferon (IFN)-gamma or TNF-alpha-activated macrophages [74]. Our previous study provided clear evidence that BA triggered oxidative stress in normal nasopharyngeal epithelial and NPC cells. The effect of BA in the induction of oxidative stress was enhanced by the acid [75]. These findings unravelled a possibility that oxidative stress provoked by the acidic gastric duodenal content may be a vital factor leading to the inflammation-induced carcinogenesis in the nasopharyngeal epithelium. It will be intriguing to investigate the relation between BA and proinflammatory or anti-inflammatory factors in the context of direct exposure of the nasopharyngeal epithelial cells to the refluxate.

In addition, BA-induced apoptosis has been suggested to be a possible mechanism underlying the pathogenesis of Barrett’s oesophagus, oesophagus adenocarcinoma and colon cancer [76–78]. Chromosomal cleavage is a hallmark of apoptosis. Initially, the chromosomal DNA is being cleaved and detached from their binding sites on the nuclear scaffold. The release of rosettes and loops of chromatin produces the high-molecular-weight (HMW) DNA of 200 to 300 and 30 to 50 kbp, respectively [79–81]. In the later stage of apoptosis, the HMW DNA is further degraded into internucleosomal DNA fragments of 180 to 200 bp [82, 83]. In our previous study, we demonstrated that BA was able to induce apoptosis in normal nasopharyngeal epithelial and NPC cells. We further demonstrated that BA-induced apoptosis resulted in chromosome breakages within the *AF9* gene. These chromosome breakages were abolished by the caspase-3 inhibitor. Given that caspase-3 is the primary activator of caspase-activated DNase (CAD), our findings suggested that CAD may play an important role in mediating the chromosomal cleavages during BA-induced apoptosis [75].

It has been observed that the apoptotic nuclease CAD is closely associated with the nuclear matrix in the cells undergoing apoptosis [84]. Chromosomal DNA binds to the nuclear matrix through matrix association region/scaffold attachment region (MAR/SAR) [85]. It is plausible that when CAD cleaves the chromosomal DNA, it potentially cleaves at MAR/SAR. Thus, we hypothesised that BA-induced apoptosis may cause DNA breakages preferentially at MAR/SAR sites leading to chromosome rearrangement in NPC. Our study focuses on the *AF9* gene which is located at 9p22 because 9p22 is one of the deletion hotspots in NPC [86]. In the present study, we performed in silico prediction of MAR/SAR within the *AF9* gene. We demonstrated that the *AF9* gene cleavage frequency within the SAR region was significantly higher in BA-treated cells as compared with the untreated control. By contrast, there was no significant difference in the *AF9* gene cleavage frequency within the non-SAR region between BA-treated and untreated control cells. Our results suggest a role for MAR/SAR in defining the positions of chromosomal breakages mediated by BA-induced apoptosis.

## Methods

### Cell lines and chemicals

NP69 normal nasopharyngeal epithelial cell line was generously provided by Prof. Tsao Sai Wah (The University of Hong Kong, Hong Kong, China) and Prof. Lo Kwok Wai (The Chinese University of Hong Kong, Hong Kong, China). TWO4 NPC cell line was kindly given by Prof. Sam Choon Kook (formerly from University of Malaya, Malaysia).

Keratinocyte-SFM medium (17005–042), RPMI 1640 medium (21870–076), penicillin/streptomycin (15140–122), L-glutamine (25030–081) and fetal bovine serum (10270–098) were bought from GIBCO, Invitrogen, USA. Taurocholic acid sodium salt hydrate (T4009), sodium glycochenodeoxycholate (G0759), glycocholic acid sodium (G2878), sodium deoxycholate (D2510), sodium glycodeoxycholate (G6132), dibasic sodium phosphate (255793) and citric acid (251275) were purchased from Sigma, USA. Ammonium acetate (101116) was procured from Merck, Germany. Chloroform (288306) and isoamyl alcohol (W205702) were purchased from Sigma-Aldrich, Malaysia. Phenol (UN2821) and sodium dodecyl sulfate (SDS) (151–21-3) were obtained from Amresco, USA. Phusion High-Fidelity DNA Polymerase (F-530 L) was bought from Finnzymes, Finland. *Bam*H I (R013S), *Kpn* I (R0142S), *Nde* I (R0111S), *Hin*d III (R0104S), *Xba* I (R0145S), T4 DNA Ligase (M0202 L) and DNA Polymerase I, Large (Klenow) Fragment (M0210S) were procured from New England Biolabs (NEB), USA. dNTP mix (U1515) was purchased from Promega, USA. PCR primers were obtained from First Base Laboratories. QIAquick Gel Extraction Kit (28704) and Nucleotide Removal Kit (28304) were bought from QIAGEN, Germany.

### In silico prediction of MAR/SAR within the *AF9* gene

#### MAR/SAR recognition signature (MRS)

The *AF9* gene sequence was accessed from Ensembl database [EMBL:ENSG00000171843]. By using DNASTAR software (Lasergene, USA), MAR/SARs within the *AF9* gene were predicted by searching MAR/SAR recognition signature (MRS). MRS is a bipartite sequence strongly related to MAR/SAR [87]. MRS consists of two nucleotide motifs that are found within a distance of 200 bp. The first nucleotide motif is an 8 bp degenerate sequence, AATAAYAA. Exact match is required for this 8 bp sequence. The second nucleotide motif is a 16 bp degenerate sequence, AWWRTAANNWWGNNNC, where Y = C or T; W = A or T; R = A or G; N = A, C, G or T. One mismatch is permitted within the 16 bp sequence. The distance between these two degenerate sequences should be within 200 bp. Each sequence can be found on either Watson (W) strand or Crick (C) strand. The sequences may be overlapping or one precedes the other. When there is more than one motif of either 8 or 16 bp to be found within the constraint of the pattern, they are considered as a single MRS. Besides, when there is more than one MRS to be identified within close proximity, they are regarded as a single potential MAR/SAR site. The locations of the MRS predicted MAR/SARs were compared with the locations of the experimentally determined MAR/SARs identified in previous studies [88, 89].

We have also performed in silico prediction of MAR/SAR in the abelson murine leukaemia viral oncogene homolog 1 (*ABL)* gene by using MRS [90]. We only found one predicted MAR/SAR site which matches to the experimentally determined MAR/SAR in the *ABL* gene. However, the distance between the 8 bp sequence element and the 16 bp sequence element was found to be 248 bp. Therefore, in this study, we set the maximal distance between 8 bp sequence element and the 16 bp sequence element at 250 bp.

#### SMARTest and MAR-finder

The *AF9* gene sequence was further analysed by using two MAR/SAR prediction tools, namely, SMARTest (http://www.genomatix.de) [91] and MAR-Finder (http://genomecluster.secs.oakland.edu/marwiz/) [92].

### Cell cultures

NP69 cells were grown in Keratinocyte-SFM medium supplemented with 2% (*v*/v) heat-inactivated fetal bovine serum, 4–5 ng/ml recombinant Epidermal Growth Factor (rEGF), 40–50 μg/ml Bovine Pituitary Extract (BPE), 100 U/ml penicillin and 100 μg/ml streptomycin. TWO4 cells were grown in RPMI 1640 medium supplemented with 10% (v/v) heat-inactivated fetal bovine serum, 2 mM L-glutamine, 100 U/ml penicillin and 100 μg/ml streptomycin. Cells were cultured with 5% CO_2_ at 37 °C.

### Preparations of BA cocktail and media for BA treatment

The BA cocktail was prepared as previously described [93]. It consists of an equimolar mixture of sodium salts of deoxycholic acid, glycochenodeoxycholic acid, glycocholic acid, glycodeoxycholic acid and taurocholic acid. The concentration of each of the five bile salts was 0.02 mM for a total BA concentration of 0.1 mM. Total BA concentrations in the refluxate of patients with Barrett’s oesophagus were reported to range from 0.03 to 0.82 mM [94]. Higher concentrations of BA (as high as 7.6 mM) have also been observed in the refluxate of some patients with Barrett’s oesophagus [95]. Therefore, in this study, we used a concentration within the physiological range (0.5 mM).

In addition, it has been reported that nasopharyngeal reflux was more prevalent in CRS patients. Nasopharyngeal pH less than 5 has been observed in 76% of these nasopharyngeal reflux-related CRS patients [43]. Similarly, in a 24-h pH monitoring study, abnormal nasopharyngeal pH (nasopharyngeal pH mean was 5.6917) has been reported in patients with GORD related-chronic respiratory diseases (otitis, sinusitis, laryngitis, epiglottitis, recurrent stridor, asthma and recurrent pneumonia). A 5.8 nasopharyngeal pH was regarded as the most sensitive and specific cut-off point to show the presence of abnormal pH-metry in the patients with nasopharyngeal reflux related-chronic respiratory diseases [96]. Therefore, in our studies, the BA treatments were performed at neutral pH (pH 7.4) and acidic pH (pH 5.8). The media used for BA treatment at acidic pH were acidified to pH 5.8 with citrate phosphate buffer.

### Nested inverse polymerase chain reaction (IPCR) detection of BA-induced chromosome breaks

#### BA treatment

NP69 cells (1.5 × 10^4^) and TWO4 cells (2.5 × 10^4^) were seeded in 60 mm culture dishes and allowed to grow for 2 days. NP69 cells were left untreated or treated with 0.5 mM of BA cocktail at pH 7.4 and pH 5.8 for 1 h. TWO4 cells were left untreated or treated with 0.5 mM of BA cocktail at pH 7.4 and pH 5.8 for 3 h.

#### Genomic DNA extraction

After exposure, the cells were subjected to genomic DNA extraction by using phenol/chloroform/isoamyl alcohol extraction method as previously described [97].

#### Manipulation of genomic DNA for the AF9 SAR region

In order to prepare the DNA for nested IPCR, several manipulation steps were carried out as previously described [97]. The simplified manipulation steps were illustrated in Additional file 1. Firstly, digestion with 100 U of *Bam*H I (RE1 in Additional file 1) was performed. This was followed by Klenow fill-in, ligation and ethanol precipitation. The DNA was either digested with *Kpn* I (RE2 in Additional file 1) or *Nde* I (RE3 in Additional file 1). The digested DNA was cleaned up by using QIAGEN QIAquick Nucleotide Removal Kit according to the manufacturer’s protocol.

#### Nested IPCR for the AF9 SAR region

The reaction for nested IPCR consisted of 1X of HF buffer (containing 1.5 mM of MgCl_2_), 0.5 μM of each reverse primer and forward primer, 200 μM of dNTP mix, 0.4 U of Phusion High-Fidelity DNA Polymerase, and 200 ng of DNA template. Sterile ultrapure water was used to replace DNA template in the negative control. The cycle condition used in the first round was as below: 30 s of 98 °C for 1 cycle (initial denaturation), followed by 30 cycles of 98 °C for 10 s (denaturation), 69 °C for 30 s (annealing), 72 °C for 15 s (extension), followed by 1 cycle of 72 °C for 10 min (final extension). Two μl of 5-fold diluted first round IPCR product was used as DNA template for the second round. The cycle condition used in the second round was similar to that in the first round, except that the annealing temperature was 57 °C. The primers used in the first round of IPCR were 5’-ATTCTAGACCCCAAAAAATTCTCAG-3′ (reverse) and 5’-CTCTTAATGCCACTGCCATGA-3′ (forward), whereas the primers used in the second round were 5’-CATATCCTTTTCATACCTGG-3′ (reverse) and 5’-ATTGGTGTCAATCAAATGC-3′ (forward). The IPCR amplifications were performed by using a Veriti 96 Well Thermal Cycler (Applied Biosystems, USA).

#### Manipulation of genomic DNA and nested IPCR for the AF9 non-SAR region

The manipulation steps were similar with that of the SAR region, except that *Hin*d III (RE2 in Additional file 1) and *Xba* I (RE3 in Additional file 1) were used for the *AF9* non-SAR region instead of *Kpn* I and *Nde* I. The cycle condition used in the first round of IPCR was as below: 30 s of 98 °C for 1 cycle (initial denaturation), followed by 30 cycles of 98 °C for 10 s (denaturation), 64 °C for 30 s (annealing), 72 °C for 22 s (extension), followed by 1 cycle of 72 °C for 10 min (final extension). Two μl of 5-fold diluted first round IPCR product was used as DNA template for the second round. The cycle condition of the second round was similar to that of the first round, except that the annealing temperature was 63 °C and the extension time was 15 s. The primers used for the first round of IPCR were 5′- TACCAAACATTTTGAGTCCTACAG-3′ (reverse) and 5′- GGCATTCAGGTGAGTAGTTTATTC-3′ (forward), whereas the primers used in the second round were 5’-AGCAGTAGACTTTTGTAACCTCAC-3′ (reverse) and 5′- AGGGGATGACTTTTCTTCAATC-3′ (forward).

### Agarose gel electrophoresis and DNA sequencing of the IPCR bands

To visualise the *AF9* cleaved fragments, the IPCR products were loaded on 1% agarose gel and stained with ethidium bromide. The IPCR bands representing the *AF9* cleaved fragments were excised. The IPCR products were extracted by using QIAGEN QIAquick Gel Extraction Kit and sequenced. The sequencing results were then annotated by blasting the human genome database (Nucleotide BLAST, http://blast.ncbi.nlm.nih.gov/Blast.cgi). The breakpoints of the *AF9* cleaved fragments were identified by aligning the sequencing data with the *AF9* gene sequence retrieved from Ensembl database [EMBL:ENSG00000171843]. This was done by using Seqman DNASTAR software (Lasergene, USA). A genomic map depicting the positions of the chromosome breaks in relation to the chromatin structure was constructed.

### Quantification of gene cleavage frequency

The IPCR assays were carried out in two to four sets per experiment. Each set of IPCR assay consisted of three to six replicates per cell sample. The gene cleavage frequencies represent the average number of the *AF9* cleaved fragments detected in two to three independent experiments.

### Prediction of topo II consensus sites

Topo II consensus sites were predicted as previously described [98, 99]. Topo II consensus site was proposed to be associated with an 18 bp DNA sequence, 5’RNYNNCNNGYNGKTNYNY 3′. There are ten specific and eight non-specific nucleotides in this sequence. One mismatch is allowed for the ten of the specific nucleotides in one DNA strand whereas five mismatches are allowed in the opposite strand.

### Statistical analysis

Data were presented as means with standard deviation (SD). Student’s *t*-test was used to evaluate the significance of differences between the untreated control and the treated groups in flow cytometric analyses and IPCR assays. Mann-Whitney *U* test was used to evaluate the significance of differences in cleavage frequencies between the SAR region and non-SAR region. All statistical tests were two-sided. Differences were considered statistically significant at *p*-value < 0.05.

## Results

### In silico prediction of MAR/SAR

#### MAR/SAR recognition signature (MRS)

This study targeted the *AF9* gene located at 9p22. The *AF9* gene is 280,880 bp in length [EMBL:ENSG00000171843]. It consists of 10 exons (Additional file 2). Potential MAR/SAR sites in the *AF9* were predicted by MAR/SAR recognition signature (MRS). MRS was proposed to be strongly associated with MAR/SAR [87]. Forty-one MRS predictions were found in the *AF9* gene. These 41 MRS correspond to 29 MAR/SAR candidates, as some of the MRSs which cluster within close proximity were regarded as a single potential MAR/SAR site.

Table 1 shows the nucleotide positions of the MRSs with their sequence composition, relative orientation, distance between the two sequence elements and location of the MRSs in the exon or intron of the *AF9* gene. Intron 2 with 164 kb in length is the largest intron of the *AF9* gene. Almost half of the MAR/SAR sites (14 out of the 29 predicted MAR/SARs) were found in this largest intron (MAR/SARs 2–15 in Table 1). Both intron 3b (MAR/SARs 17–21 in Table 1) and intron 4 (MAR/SARs 22–26 in Table 1) were found to contain five MAR/SAR sites. Two potential MAR/SAR sites (MAR/SARs 27–28 in Table 1) were identified in intron 7. Intron 1 (MAR/SAR 1 in Table 1), intron 3a (MAR/SAR 16 in Table 1) and intron 9 (MAR/SAR 29 in Table 1) were all found to contain one MAR/SAR site.

**Table 1:** MRS-predicted MAR/SAR sites within the *AF9* gene

MAR/SAR	MRS	AATAAYAA (8 bp)	Nucleotide position	AWWRTAANNWWGNNNC (16 bp)	Nucleotide position	Distance (bp)	Location in exon/intron
1	1	AATAATAA (C)	915–922	ATAATAATAAAAGCCC (C)	916–931	Overlap	Intron 1
		AATAATAA (C)	918–925			Overlap	
2	2	AATAATAA (W)	5694–5701	ATAGTAAGGATGGCTG (W)	5636–5651	42	Intron 2
3	3	AATAACAA (W)	10,561–10,568	AAAATAACAAAGGAAG (W)	10,555–10,570	Overlap	Intron 2
4	4	AATAATAA (W)	26,420–26,427	AATATTATTATGGGTC (W)	26,366–26,381	38	Intron 2
5	5	AATAACAA (W)	47,627–47,634	AAAGTAAACTGGAAAC (C)	47,851–47,866	− 216	Intron 2
6	6	AATAATAA (W)	56,227–56,234	AAAATAATAATAATAC (W)	56,224–56,239	Overlap	Intron 2
		AATAATAA (W)	56,230–56,237			Overlap	
7	7	AATAACAA (W)	93,895–93,902	AAAATCATCTTGGGAC (W)	94,045–94,060	−142	Intron 2
8	8	AATAATAA (C)	108,635–108,642	AAAATAATAAAAACCC (C)	108,633–108,648	Overlap	Intron 2
9–1	9	AATAATAA (C)	112,368–112,375	ATAATAACATTTTACC (C)	112,369–112,384	Overlap	Intron 2
9–2	10	AATAATAA (C)	113,271–113,278	AAAATAATAATTGTAC (C)	113,269–113,284	Overlap	Intron 2
10	11	AATAACAA (W)	117,606–117,613	ATTGGAATGTAGAAAC (W)	117,722–117,737	−108	Intron 2
11–1	12	AATAACAA (C)	128,355–128,362	AATATAATCTAATTGC (W)	128,593–128,608	−230	Intron 2
11–2	13	AATAACAA (C)	129,941–129,948	AAAATAAGTTTCCAGC (W)	129,838–129,853	87	Intron 2
						126	
		AATAATAA (C)	129,980–129,987				
12	14	AATAATAA (W)	136,182–136,189	ATAATAATAAAATCAC (W)	136,176–136,191	Overlap	Intron 2
13	15	AATAATAA (C)	139,902–139,909	AATATAATGAATATCC (C)	139,927–139,942	−17	Intron 2
						−14	
		AATAATAA (C)	139,905–139,912			−11	
						−8	
		AATAATAA (C)	139,908–139,915				
		AATAATAA (C)	139,911–139,918				
14	16	AATAATAA (W)	151,857–151,864	TTTATAAACTTGTTTC (C)	151,672–151,687	169	Intron 2
15	17	AATAATAA (W)	158,593–158,600	AAAATAAAAAAGAGCT (C)	158,541–158,546	46	Intron 2
16	18	AATAATAA (W)	170,529–170,536	AAAATAATAAATACGC (W)	170,523–170,538	Overlap	Intron 3a
		AATAACAA (W)	170,619–170,626			80	
17–1	19	AATAATAA (W)	178,634–178,641	ATAATAAATATGAATA (W)	178,638–178,653	Overlap	Intron 3b
17–2	20	AATAATAA (W)	179,140–179,147	AAAAGAACTAAGGTAC (W)	179,378–179,393	−230	Intron 3b
18	21	AATAATAA (C)	183,173–183,180	AAGGTAAATTAGCAGC (W)	182,936–182,951	221	Intron 3b
				ATAATAATAATGTTCT (C)	183,171–183,186	Overlap	
				ATAATAATGTTCTACC (C)	183,174–183,189	Overlap	
19	22	AATAATAA (W)	191,323–191,330	ATTATAAGAAAAATTC (W)	191,065–191,080	242	Intron 3b
				ATAATAAAAATGTTAT (C)	191,076–191,091	231	
				ATAATGATCAAGTACC (C)	191,548–191,563	− 217	
20–1	23	AATAATAA (W)	194,511–194,518	AAAATAAGAAAACATC (W)	194,333–194,348	162	Intron 3b
				AATATAATTATGCTAA (W)	194,753–194,768	−234	
20–2	24	AATAATAA (C)	195,198–195,205	AATATAAAATTGCAAG (W)	195,275–195,290	−69	Intron 3b
21	25	AATAATAA (W)	200,774–200,781	AAAATAATAAAGCCAT (W)	200,768–200,783	Overlap	Intron 3b
22	26	AATAATAA (W)	215,368–215,375	ATAATAATAATAATAC (W)	215,365–215,380	Overlap	Intron 4
						Overlap	
		AATAATAA (W)	215,371–215,378				
						Overlap	
		AATAATAA (W)	215,374–215,381				
	27	AATAACAA (C)	215,941–215,948	AAAATAAAACTGACTC (C)	215,781–215,796	144	Intron 4
				ATAATTACATAGACAC (W)	216,113–216,128	−164	
23	28	AATAATAA (C)	227,848–227,855	ATAATAATAATGAAAG (C)	227,849–227,864	Overlap	Intron 4
		AATAATAA (C)	227,851–227,858			Overlap	
24–1	29	AATAATAA (W)	236,308–236,315	ATAATAAGTTATAGGC (W)	236,299–236,314	Overlap	Intron 4
		AATAATAA (C)	236,343–236,350			28	
24–2	30	AATAACAA (W)	237,611–237,618	AAAATAACAAAATGTC (W)	237,605–237,620	Overlap	Intron 4
				AATGTAAGCAATATCC (W)	237,817–237,832	−198	
24–3	31	AATAATAA (W)	238,001–238,008	AATGTAAGCAATATCC (W)	237,817–237,832	168	Intron 4
				AAAGTATTGTAGACCC (C)	237,983–237,998	2	
				AAAATAATAAAGGGGT (W)	237,995–238,010	Overlap	
24–4	32	AATAATAA (W)	239,050–239,057	TATATAATAAAGTGAC (C)	238,794–238,809	240	Intron 4
25–1	33	AATAATAA (C)	246,588–246,595	ATAATAATGAAGAAAG (C)	246,610–246,625	−14	Intron 4
25–2	34	AATAACAA (C)	247,561–247,568	ATTGTAATATTGATTG (C)	247,587–247,602	−18	Intron 4
				AATATTACAATGAATC (W)	247,582–247,597	−13	
				TTTATAAATTAGGGAC (W)	247,758–247,773	−189	
26–1	35	AATAATAA (C)	251,344–251,351	AAAGTAAATAAAAAAC (W)	251,265–251,280	63	Intron 4
26–2	36	AATAATAA (C)	252,893–252,900	AATGAAAGGAAGAGCC (W)	252,636–252,651	241	Intron 4
				ATAATAATAATGAAAA (C)	252,891–252,906	Overlap	
27	37	AATAATAA (W)	262,940–262,947	ATAATAAACTACCATC (W)	262,931–262,946	Overlap	Intron 7
				ATAATAATAAACTACC (W)	262,934–262,949	Overlap	
				AAAATAAACTATTTTC (C)	263,198–263,213	−250	
28–1	38	AATAATAA (C)	265,768–265,775	AATATAATCTTGAACG (C)	265,612–265,627	140	Intron 7
28–2	39	AATAATAA (C)	267,133–267,140	AAAATAAAAATATGCC (C)	266,963–266,978	154	Intron 7
				AATAAAAATATGCCCC (C)	266,965–266,980	152	
				ATAATAAGGCTGGGAC (C)	267,134–267,149	Overlap	
				ATTTTAAGAATGAGTC (W)	267,205–267,220	−64	
				AAGATAAATTAGGTCC (C)	267,317–267,332	−176	
28–3	40	AATAATAA (C)	267,569–267,576	AAGATAAATTAGGTCC (C)	267,317–267,332	236	Intron 7
29	41	AATAACAA (W)	273,528–273,535	AAAAAAAAATTGTAAC (W)	273,593–273,608	−57	Intron 9

MRSs with their nucleotide positions, sequence composition, relative orientation (C, Crick strand and W, Watson strand), distance between the two sequence motifs, and location in the exon or intron of the *AF9* gene are shown. A negative distance indicates that 8 bp sequence element precedes the 16 bp sequence element

**Fig. 1:**
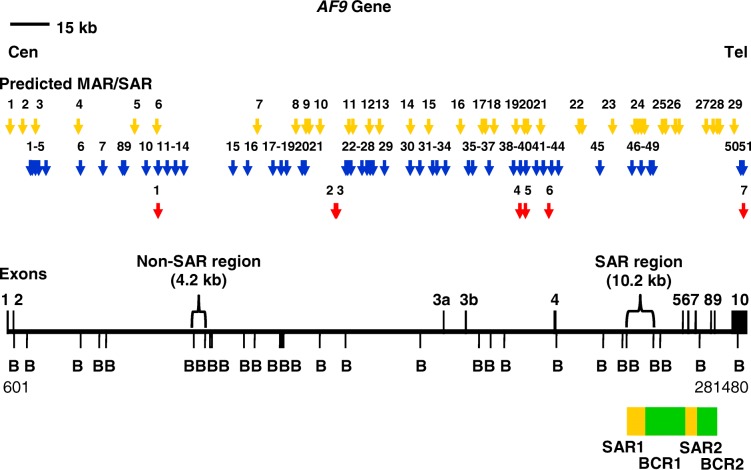
Potential MAR/SAR sites within the *AF9* gene. The *AF9* genomic map from nucleotide positions 601–281,480 is depicted [EMBL:ENSG00000171843]. The locations of exons 1 to 10 and *Bam*H I (B) restriction sites are shown. Green boxes represent the two previously identified patient BCRs which were indicated as BCR1 and BCR2 [88]. Yellow boxes represent the two experimentally verified MAR/SARs (denominated as SAR1 and SAR2) reported in the previous study [88]. Yellow, blue and red arrows represent the potential MAR/SAR sites predicted in our present study by using MRS, SMARTest and MAR-Finder, respectively. Based on the previous reports and the in silico prediction in the present study, a SAR region (contains MAR/SAR) and a non-SAR region (does not contain MAR/SAR) were determined to be the regions of study

Figure 1 (yellow arrows) shows the distribution of the MRS predicted MAR/SAR sites in the *AF9* gene. Two experimentally determined MAR/SARs have been reported in the previous study. These two MAR/SARs were designated as SAR1 and SAR2. SAR1 is located in intron 4. SAR2 spans from exons 5 to 7 [88]. Based on the locations of the previously reported biochemically defined MAR/SAR sites and the presently predicted MAR/SAR sites, a SAR region (contains MAR/SAR) and a non-SAR region (does not contain MAR/SAR) were determined as the region of our study (Fig. 1). The *AF9* SAR region contains one MRS- predicted MAR/SAR site (MAR/SAR 24) which matches to the biochemically defined SAR1. By contrast, the *AF9* non-SAR region is a region which does not contain any biochemically extracted MAR/SAR or MRS- predicted MAR/SAR.

#### SMARTest and MAR-finder

The *AF9* sequence was further analysed by using two MAR/SAR prediction programs, namely SMARTest (http://www.genomatix.de) and MAR-Finder (http://genomecluster.secs.oakland.edu/marwiz/). The distribution of MAR/SARs predicted by SMARTest and MAR-Finder were shown in Fig. 1 (blue and red arrows, respectively). Table 2 shows the nucleotide position of the potential MAR/SAR sites identified by SMARTest. There were 51 MAR/SARs predicted within the *AF9* gene. There was no MAR/SAR predicted within the non-SAR region. The closest MAR/SAR at its 5′ site locates at coordinates 66,686–67,255 (MAR/SAR 14 in Table 2) while the closest MAR/SAR at its 3′ site locates at coordinates 85,486–85,800 (MAR/SAR 15 in Table 2). On the other hand, there were four MAR/SARs predicted within the SAR region. These four MAR/SARs locate at coordinates 237,321–237,855, 240,926–241,315, 244,311–244,710 and 245,416–245,850 (MAR/SARs 46–49 in Table 2). The locations of these four SMARTest- predicted MAR/SARs match to the experimentally determined SAR1 (Fig. 1).

**Table 2:** SMARTest-predicted MAR/SAR sites within the *AF9* gene

MAR/SAR	Start	End	Length in bp	Location in exon/intron
1	8376	8750	375	Intron 2
2	9066	9515	450	Intron 2
3	10,231	10,755	525	Intron 2
4	11,381	11,900	520	Intron 2
5	13,726	15,420	1695	Intron 2
6	27,451	27,885	435	Intron 2
7	35,906	36,320	415	Intron 2
8	43,476	43,805	330	Intron 2
9	44,151	44,570	420	Intron 2
10	52,401	52,710	310	Intron 2
11	56,866	57,420	555	Intron 2
12	60,376	60,865	490	Intron 2
13	63,716	64,080	365	Intron 2
14	66,686	67,255	570	Intron 2
15	85,486	85,800	315	Intron 2
16	91,121	91,480	360	Intron 2
17	100,546	101,035	490	Intron 2
18	103,826	104,175	350	Intron 2
19	105,881	106,415	535	Intron 2
20	111,831	112,155	325	Intron 2
21	112,851	113,415	565	Intron 2
22	128,371	128,860	490	Intron 2
23	129,216	129,590	375	Intron 2
24	130,431	130,740	310	Intron 2
25	134,286	135,320	1035	Intron 2
26	136,616	136,965	350	Intron 2
27	137,456	138,445	990	Intron 2
28	138,806	139,170	365	Intron 2
29	143,206	143,575	370	Intron 2
30	152,956	153,395	440	Intron 2
31	156,626	156,975	350	Intron 2
32	161,516	162,005	490	Intron 2
33	163,066	163,370	305	Intron 2
34	166,416	166,795	380	Exon 3a/intron 3a
35	175,101	175,570	470	Intron 3a
36	176,771	177,165	395	Intron 3a
37	183,136	183,455	320	Intron 3a
38	192,376	192,700	325	Intron 3a
39	194,406	195,730	1325	Intron 3a
40	197,086	197,470	385	Intron 3a
41	201,111	201,490	380	Intron 3a
42	203,651	203,980	330	Intron 3a
43	206,656	207,480	825	Intron 3a
44	209,211	210,115	905	Exon 4/intron 4
45	225,211	225,550	340	Intron 4
46	237,321	237,855	535	Intron 4
47	240,926	241,315	390	Intron 4
48	244,311	244,710	400	Intron 4
49	245,416	245,850	435	Intron 4
50	278,816	279,335	520	Exon 10
51	279,716	280,100	385	Exon 10

**Fig. 2:**
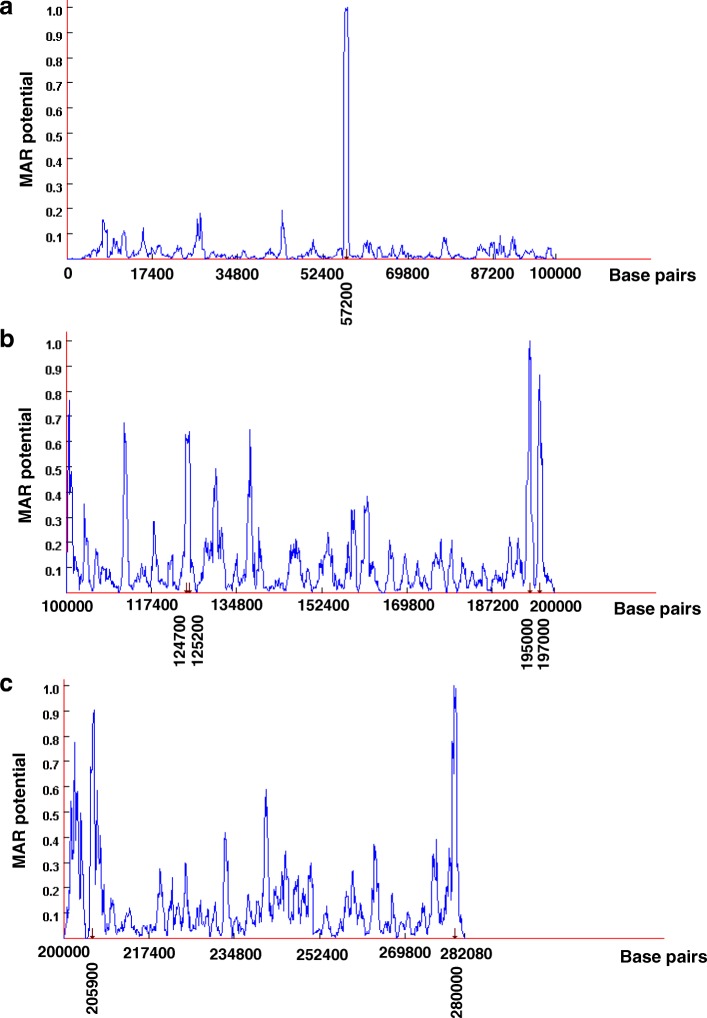
MAR-Finder predictions within the *AF9* gene. (a) Coordinates 0–100,000 (b) Coordinates 100,000–200,000 (**c**) Coordinates 200,000–282,080 [Ensembl:ENSG00000171843]. There were seven MAR/SARs predicted within the *AF9* gene. These seven potential MAR/SARs locate at 57200 (**a**), 124,700, 125,200, 195,000, 197,000 (**b**), 205,900 and 280,000 (**c**)

Figures 2 a, b and c show the MAR-Finder predicted MAR/SARs within the *AF9* gene for coordinates 0–100,000, 100,000–200,000 and 200,000–282,080, respectively [Ensembl:ENSG00000171843]. MAR-Finder predicted seven MAR/SAR sites within the *AF9* gene. These seven potential MAR/SARs locate at 57200 (Fig. 2 a), 124,700, 125,200, 195,000, 197,000 (Fig. 2 b), 205,900 and 280,000 (Fig. 2 c). There was no MAR/SAR predicted within the SAR region or the non-SAR region.

### IPCR detection of chromosome breaks within the *AF9* SAR and non-SAR regions upon BA treatment

Nested IPCR was employed to investigate if BA-induced apoptosis does lead to cleavage in the *AF9* SAR and non-SAR regions. Genomic DNA extraction and subsequent IPCR were performed on BA-treated NP69 and TWO4 cells. Based on the primers position, if there is no chromosomal breakage detected, the IPCR product for the SAR region of the *AF9* gene will be 944 bp (~ 950 bp), while for the non-SAR region of the *AF9* gene, the IPCR product will be 956 bp (~ 950 bp). If there is any chromosomal breakage detected, for both SAR and non-SAR regions, IPCR products of less than 950 bp will be obtained.

**Fig. 3:**
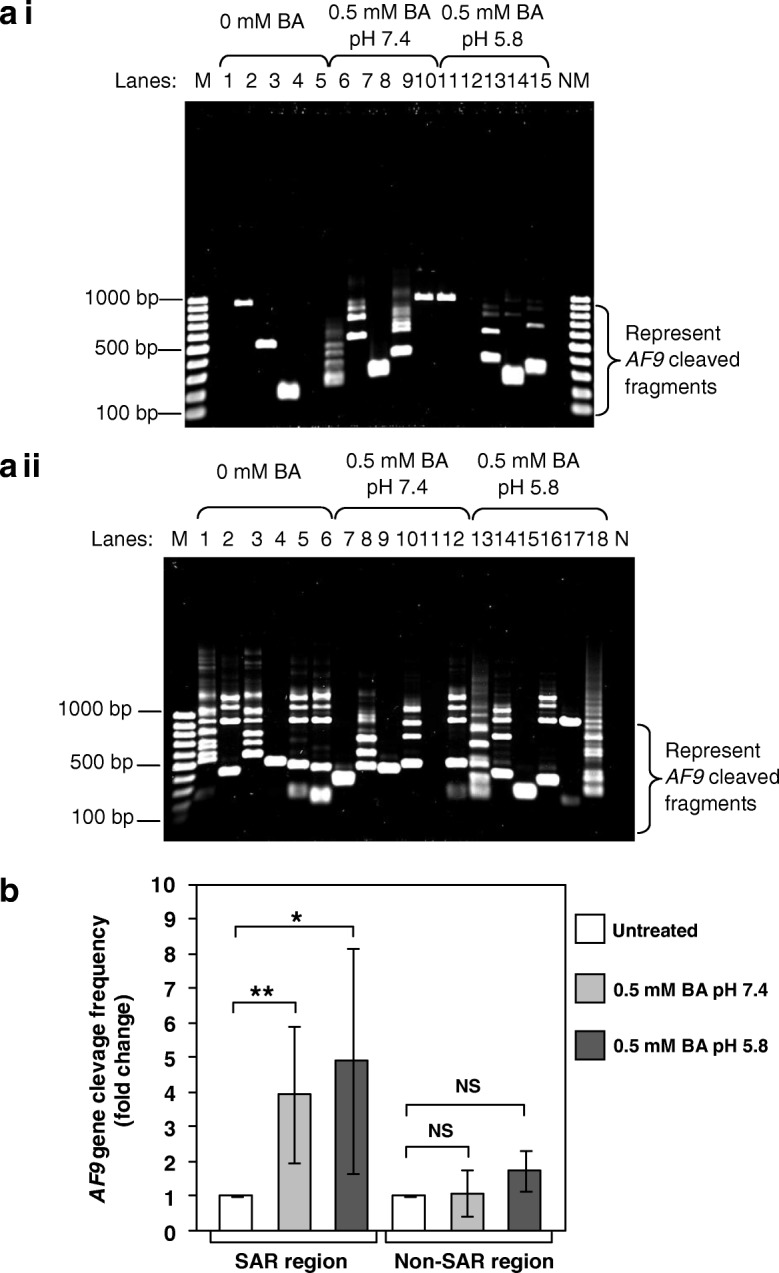
Identification of chromosome breaks in BA-treated NP69 cells. IPCR was employed to identify the *AF9* gene cleavages in NP69 cells after exposed to BA. **a** Representative gel picture showing the *AF9* gene cleavages identified by IPCR within: (**a i**) SAR region (**a ii**) Non-SAR region. NP69 cells were left untreated (**a i**, Lanes 1–5; **a ii**, Lanes 1–6) or treated for 1 h with 0.5 mM of BA at pH 7.4 (**a i**, Lanes 6–10; **a ii**, Lanes 7–12) and pH 5.8 (**a i**, Lanes 11–15; **a ii**, Lanes 13–18). Genomic DNA extraction and nested IPCR were performed as described in “Methods” section. The side bracket represents the IPCR bands derived from the *AF9* cleaved fragments. M: 100 bp DNA marker. N: negative control for IPCR. **b** The average number of the *AF9* gene cleavages identified in BA-treated NP69 cells. Data are expressed as means and SDs of two independent experiments. Each experiment consisted of two to four sets of IPCR carried out in three to six replicates per set for each cell sample. Values are expressed as fold change normalised to the value of the untreated control. The differences between untreated control and treated groups were compared by using Student’s *t* test, * *p* < 0.05, ** *p* < 0.01. NS, no significant difference

For the SAR region, NP69 cells treated with BA at pH 7.4 (Lanes 6–9) and pH 5.8 (Lanes 13–15) showed numerous IPCR bands of less than 950 bp (Fig. 3a i). These bands represent the *AF9* gene cleavages within the SAR region. Two IPCR bands were also detected in the untreated NP69 cells (Lanes 3 and 4). By using flow cytometric analyses, we have demonstrated that untreated control contained a small percentage of apoptotic cells [75]. The background might be contributed by endogenous DNA breaks which occur in these dying cells. As shown in Fig. 3b left columns, the *AF9* gene cleavage frequency detected in NP69 cells treated with BA at pH 7.4 was approximately 3.9-fold higher than that in untreated NP69 cells (*p*-value = 0.015). It was 4.9-fold higher than that of untreated control in cells exposed to BA at pH 5.8 (*p*-value = 0.032).

As for the non-SAR region, untreated NP69 cells (Lanes 1–6), and NP69 cells treated with BA at pH 7.4 (Lanes 7–10 and 12) and pH 5.8 (Lanes 13–18) showed numerous IPCR bands of less than 950 bp (Fig. 3a ii). These bands represent the *AF9* gene cleavages within the non-SAR region. There was no significant difference in the cleavage frequency between untreated NP69 cells and NP69 cells treated with BA at pH 7.4 (*p*-value = 0.807) or pH 5.8 (*p*-value = 0.086) (Fig. 3b right columns).

**Fig. 4:**
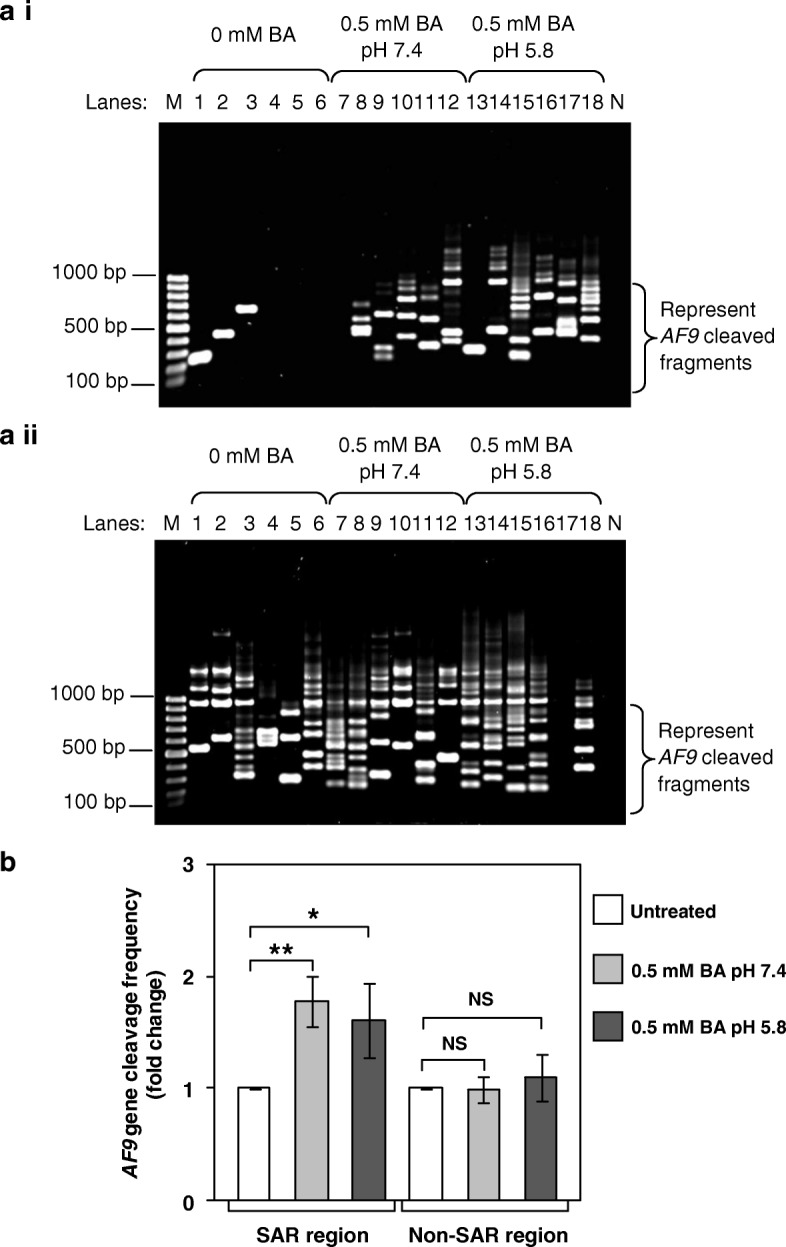
Identification of chromosome breaks in BA-treated TWO4 cells. Genomic DNA was extracted from BA-treated TWO4 cells for nested IPCR as described in “Methods” section. **a** Representative gel picture showing the *AF9* gene cleavages in BA-treated TWO4 cells detected within: (**a i**) SAR region (**a ii**) Non-SAR region. TWO4 cells were left untreated (Lanes 1–6) or treated for 3 h with 0.5 mM of BA at pH 7.4 (Lanes 7–12) and pH 5.8 (Lanes 13–18). The IPCR bands derived from the *AF9* cleaved fragments were indicated by the side bracket. M: 100 bp DNA ladder. N: Negative control for IPCR. **b** The average number of *AF9* gene cleavages detected by IPCR. Data represents means and SDs of three independent experiments. Each experiment consisted of at least two sets of IPCR assays performed in five to six replicates per set for each cell sample. Values are expressed as fold change normalised to the value of the untreated control. The differences between untreated control and treated groups were compared by using Student’s *t* test, * *p* < 0.05, ** *p* < 0.01. NS, no significant difference

Similar results were observed in TWO4 cells. Numerous IPCR bands of less than 950 bp were detected in TWO4 cells after treatment with BA at pH 7.4 (Fig. 4a i, Lanes 8–12) and pH 5.8 (Fig. 4a i, Lanes 13–18). These bands represent cleaved *AF9* gene within the SAR region. A few IPCR bands were also detected in the untreated TWO4 cells (Fig. 4a i, Lanes 1–3) which might be due to spontaneous DNA breaks. The *AF9* gene cleavage frequencies of TWO4 cells treated with 0.5 mM of BA at neutral pH and acidic pH are 1.8-fold (*p*-value = 0.004) and 1.6-fold (*p*-value = 0.036) higher than that of the untreated control, respectively (Fig. 4b left columns).

As for the non-SAR region, numerous IPCR bands of less than 950 bp were detected in untreated TWO4 cells (Fig. 4a ii, Lanes 1–6), and TWO4 cells treated with BA at pH 7.4 (Fig. 4a ii, Lanes 7–12) and pH 5.8 (Fig. 4a ii, Lanes 13–16 and 18). These bands represent cleaved *AF9* gene within the non-SAR region. As summarised in the bar chart in Fig. 4b (right columns), there was no significant difference in the cleavage frequency of the *AF9* non-SAR region between untreated TWO4 cells and TWO4 cells treated with BA at pH 7.4 (*p*-value = 0.903) or pH 5.8 (*p*-value = 0.429).

These findings clearly demonstrate that MAR/SAR mediates the gene cleavages in BA-induced apoptosis in NP69 and TWO4 cells at both neutral and acidic pH. However, in both NP69 and TWO4 cells, there were obviously more cleavage bands detected in the non-SAR region (Figs. 3a ii and 4a ii) as compared with the SAR region (Figs. 3a i and 4a i). Table 3 shows the differences in the cleavage frequencies between SAR and non-SAR regions. The cleavage frequencies of the non-SAR region detected in untreated NP69 and TWO4 cells were 4.0-fold (*p*-value = 0.001) and 5.7-fold (*p*-value < 0.001) higher than those of the SAR region, respectively. It is possible that other chromatin structures may have contributed to DNA fragility of the *AF9* non-SAR region. In addition to MAR/SAR sequence, repeat elements and topoisomerase II (topo II) cleavage site have also been well implicated in mediating chromosome breaks [88, 89, 100]. Hence, this prompted us to investigate the possibility of repeat elements and topo II cleavage site in contributing to DNA fragility of the *AF9* non-SAR region.

**Table 3:** Cleavage frequencies detected in the *AF9* SAR and non-SAR regions

Cell samples	SAR region		Non-SAR region	
	Mean	SD	Mean	SD
NP69 cells				
Untreated	0.455	0.510	1.818	1.680
1 h 0.5 mM BA, pH 7.4	1.591	2.015	1.864	1.935
1 h 0.5 mM BA, pH 5.8	1.864	1.833	3.045	2.126
TWO4 cells				
Untreated	0.830	1.108	4.710	2.312
1 h 0.5 mM BA, pH 7.4	1.780	1.791	4.830	1.880
1 h 0.5 mM BA, pH 5.8	1.730	1.957	4.960	2.510

### Identification of repeat elements

By using CENSOR program (https://www.girinst.org/censor/), repeat elements were identified in the *AF9* gene. Tables 5 and 6 show the repeat elements identified within the SAR and non-SAR regions, respectively. Eighteen repeat elements were identified within the 10.2 kb SAR region (Table 4). The overall content of repeat elements in the SAR region is 13.81%. Only one out of these 18 repeat elements is found within the amplified region. ERE2_EH (coordinates 245,627–245,728, 102 bp in length) is the only one repeat element identified within the amplified region. This repeat element occupies 11% (102 bp) of the amplified SAR region (944 bp).

**Table 4:** Repeat elements identified in the *AF9* SAR region by CENSOR program

Repeat elements		Nucleotide position		Length (bp)
Name	Class	From	To	
TWIFB1	DNA/hAT	236,920	236,987	68
MER20	DNA/hAT	237,423	237,476	54
hAT-80_HM	DNA/hAT	237,491	237,548	58
CR1-8_HM	NonLTR/CR1	237,594	237,636	43
L1ME4A	NonLTR/L1	237,637	237,719	83
GYPSY16-I_AG	LTR/Gypsy	238,883	238,925	43
MIR	NonLTR/SINE	239,516	239,716	201
ZAPHOD	DNA	239,786	239,871	86
Polinton-1_XT	DNA/Polinton	241,267	241,318	52
Hoyak1	DNA/hAT	241,475	241,555	81
L4	NonLTR/RTEX	241,769	241,847	79
ATCOPIA38_I	LTR/Copia	242,176	242,276	101
BGLII_LTR	ERV/ERV2	242,849	242,893	45
L1-1_ET	NonLTR/L1	242,989	243,024	36
ERV1–4-EC_I	ERV/ERV1	243,397	243,483	87
hATw-2_SP	DNA/hAT	244,480	244,530	51
CHARLIE7	DNA/hAT	244,901	245,043	143
ERE2_EH	Interspersed_Repeat	245,627	245,728	102
Total repeat elements		18		
Total length of repeat elements		1413 bp		
Length of the AF9 SAR region		10,234 bp		
Overall content of repeat elements		13. 81%		

The *AF9* SAR region is from coordinates 236,059 to 246,292 [Ensembl:ENSG00000171843]. The name, class, nucleotide position and length of the predicted repeat elements are shown. The region amplified by the reverse primer (AF9 236,211 R) is from coordinates 236,059 to 236,211 while the region amplified by the forward primer (AF9 245,507 F) is from coordinates 245,507 to 246,292. The amplified SAR region contains one repeat element, namely ERE2_EH (at coordinates 245,627–245,728)

**Fig. 5:**
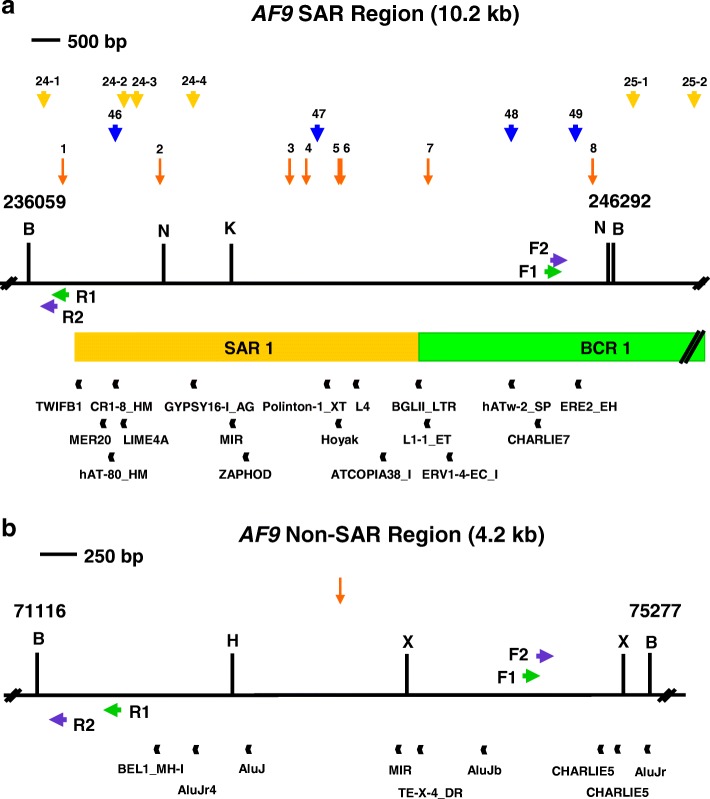
The repeats and topo II sites identified within the SAR and non-SAR regions. **a** The SAR region. The SAR region that is bordered by the two *Bam*H I sites is 10.2 kb in length (from coordinates 236,059 to 246,292). Green box represents the previously identified patient BCR which is indicated as BCR1. Yellow box shows the previously experimentally isolated MAR/SAR which is indicated as SAR1 [88]. Yellow and blue arrows represent the potential MAR/SARs predicted in this study by using MRS and SMARTest, respectively. Orange arrows represent the predicted topo II consensus sites. Green arrows represent the primers (R1, AF9 236,451 R and F1, AF9 245,385 F) used in the first round of nested IPCR while the purple arrows represent the primers (R2, AF9 236,211 R and F2, AF9 245,507 F) used in the second round of nested IPCR. Black boxes represent the repeat elements. *Bam*H I (B), *Kpn* I (K) and *Nde* I (N) restriction sites are shown. **b** The non-SAR region. The non-SAR region which is bordered by two *Bam*H I sites is 4.2 kb in length (from coordinates 71,116 to 75,277). Orange arrow represents the predicted topo II consensus site. Green arrows represent the primers (R1, AF9 71,653 R and F1, AF9 74,399 F) used in the first round of nested IPCR while the blue arrows represent the primers (R2, AF9 71,282 R and F2, AF9 74,494 F) used in the second round of nested IPCR. Black boxes represent the repeat elements. *Bam*H I (B), *Hin*d III (H) and *Xba* I (X) restriction sites are shown

On the contrary, nine repeat elements were identified within the 4.2 kb non-SAR region (Table 5). The overall content of repeat elements in the non-SAR region is 41.37%. Three out of these nine repeat elements are found within the amplified region. The three repeat elements identified in this region were two CHARLIE5 (coordinates 74,895–74,998, 104 bp in length and coordinates 75,006–75,169, 164 bp in length) and one AluJr (coordinates 75,192–75,466, 275 bp in length). These three repeat elements take up 57% (543 bp) of the amplified non-SAR region (956 bp). The locations of repeat elements identified within the SAR and non-SAR regions are illustrated in Fig. 5.

**Table 5:** Repeat elements identified in the *AF9* non-SAR region by CENSOR program

Repeat elements		Nucleotide position		Length (bp)
Name	Class	From	To	
BEL1_MH-I	LTR/BEL	71,936	71,999	64
AluJr4	NonLTR/SINE/SINE1	72,081	72,368	288
AluJ	Interspersed_Repeat	72,447	72,695	249
MIR	NonLTR/SINE	73,459	73,707	249
TE-X-4_DR	Interspersed_Repeat	73,708	73,761	54
AluJb	NonLTR/SINE/SINE1	74,030	74,304	275
CHARLIE5	DNA/hAT	74,895	74,998	104
CHARLIE5	DNA/hAT	75,006	75,169	164
AluJr	NonLTR/SINE/SINE1	75,192	75,466	275
*Total number of predicted repeat elements*		*9*		
*Total length of repeat elements*		*1722 bp*		
*Length of the AF9 non-SAR region*		*4162 bp*		
*Overall content of repeat elements*		*41.37%*		

The *AF9* non-SAR region is from coordinates 71,116 to 75,277 [Ensembl:ENSG00000171843]. The name, class, nucleotide position and length of the predicted repeat elements are shown. The region amplified by the reverse primer (AF9 71,282 R) is from coordinates 71,116 to 71,282 while the region amplified by the forward primer (AF9 74,494 F) is from coordinates 74,494 to 75,277. The amplified non-SAR region contains three repeat elements, namely two CHARLIE5 (at coordinates 74,895–74,998 and 75,006–75,169) and one AluJr (at coordinates 75,192–75,466)

### Prediction of topoisomerase II consensus sites

Table 6 and Fig. 5 summarise the topo II consensus sites predicted in the *AF9* SAR and non-SAR regions. We predicted eight topo II consensus sites in the *AF9* SAR region. The overall content of topo II sites in the SAR region is 1.41%. Two topo II consensus sites fall within the breakpoint cluster region, BCR1. Five topo II consensus sites were found within the biochemically identified MAR/SAR, SAR1. One topo II site was identified next to SAR1. Two out of the eight topo II consensus sites were found within the amplified region. These two topo II consensus sites occupy 3.81% of the amplified region. In contrast to the SAR region, only one topo II consensus site was predicted within the *AF9* non-SAR region. The overall content of topo II sites in the non-SAR region is 0.43%. This topo II consensus site is not located within the amplified region.

**Table 6:** Topo II consensus sites predicted in the *AF9* SAR and non-SAR regions

Topo II sites	DNA sequence 5’RNY NNCNNGYNGKTNYNY 3′	Percentage of matching nucleotides (%)		Nucleotide position of topo II sites
		W Strand	C Strand	
AF9 SAR region (nucleotide position 236,059–246,292)				
1	W: AACTCCTTGTTGGTCTTT	100	50	236,660–236,677
	C: AAAGACCAACAAGGAGTT			
2	W: AGAAAACAGACTGTAGAT	50	90	238,359–238,376
	C: ATCTACAGTCTGTTTTCT			
3	W: ATATACATGTGGGTGCCT	90	70	240,627–240,644
	C: AGGCACCCACATGTATAT			
4	W: GAAAAGCCACAGGAGGTT	50	90	240,917–240,934
	C: AACCTCCTGTGGCTTTTC			
5	W: ATTAAAATGTTGTTTTAT	90	50	241,485–241,502
	C: ATAAAACAACATTTTAAT			
6	W: ATTCTCATGCAAGTACAT	90	60	241,527–241,544
	C: ATGTACTTGCATGAGAAT			
7	W: GGATAACAGCCTGTAAAA	50	90	243,048–243,065
	C: TTTTACAGGCTGTTATCC			
8	W: ACTGACAAGTAGTGGTGT	90	50	245,924–245,941
	C: ACACCACTACTTGTCAGT			
Total number of predicted topo II sites		8		
Total length of predicted topo II sites		144 bp		
Length of the *AF9* SAR region		10,234 bp		
Overall content of topo II sites		1.41%		
AF9 non-SAR region (nucleotide position: 71116–75,277)				
1	W: ATCAACATTCAGTTGTAT	90	50	73,170–73,187
	C: ATACAACTGAATGTTGAT			
Total number of predicted topo II sites		1		
Total length of predicted topo II sites		18 bp		
Length of the *AF9* non-SAR region		4162 bp		
Overall content of topo II sites		0.43%		

### Sequencing results

To ascertain that the cleavage fragments identified in IPCR were derived from the cleaved *AF9* gene, some of the IPCR bands were excised, purified and sequenced. The sequencing results show that these fragments were all derived from the cleaved *AF9* gene. The breakpoints identified within the *AF9* SAR region in BA-treated NP69 and TWO4 are shown in Tables 7 and 8, respectively. Intriguingly, several breakpoints (at coordinates 245,509, 245,527, 245,575, 245,577, 245,594, 245,596 and 245,612) were mapped within the *AF9* region (at coordinates 245,252–245,612) which was previously reported to be involved in t(9;11)(p22;q23). This chromosome translocation resulted in the formation of mixed lineage leukaemia (*MLL*)*-AF9* fusion gene in acute lymphoblastic leukaemia (ALL) patient [GenBank:AM050804]. It is noteworthy that one of the presently identified breakpoints is identical with that identified in the ALL patient (at coordinate 245,612) [GenBank:AM050804].

**Table 7:** Breakpoints detected within the *AF9* SAR region in BA-treated NP69 cells

BA-treated NP69 cells	Breakpoint	Remarks
0.5 mM BA, pH 7.4	245,527	This chromosome break falls within the *AF9* region (at coordinates 245,252–245,612) that was previously reported to translocate with the *MLL* gene leading to the formation of the *MLL*-*AF9* fusion gene in an ALL patient [GenBank:AM050804].
	245,575	This chromosome break falls within the *AF9* region (at coordinates 245,252–245,612) that was previously reported to translocate with the *MLL* gene leading to the formation of the *MLL*-*AF9* fusion gene in an ALL patient [GenBank:AM050804].
	245,596	This breakpoint is identical with a breakpoint identified in TWO4 cells treated with BA at pH 7.4. This chromosome break falls within the *AF9* region (at coordinates 245,252–245,612) that was previously reported to be involved in the formation of the *MLL*-*AF9* fusion gene in an ALL patient. This breakpoint is three nucleotides different from that reported in cultured normal blood cells treated with VP16 (at coordinate 245,593) [101], five nucleotides different from a breakpoint detected in H_2_O_2_-treated NP69 cells (at coordinate 245,591) and six nucleotides different from that identified in H_2_O_2_-treated HK1 cells (at coordinate 245,590) [97].
	245,649	This chromosome break falls within a repeat ERE2_EH (at coordinates 245,627–245,728).
	245,725	This chromosome break falls within a repeat ERE2_EH (at coordinates 245,627–245,728).
	245,979	
0.5 mM BA, pH 5.8	245,621	This breakpoint is nine nucleotides different from that identified in an ALL patient (at coordinate 245,612) [GenBank:AM050804].
	245,699	This breakpoint is four nucleotides different from that reported in H_2_O_2_-treated NP69 cells (at coordinate 245,703) [97]. This chromosome break falls within a repeat ERE2_EH (at coordinates 245,627–245,728).
	245,708	This breakpoint is five nucleotides different from that reported in H_2_O_2_-treated NP69 cells (at coordinate 245,703) [97]. This chromosome break falls within a repeat ERE2_EH (at coordinates 245,627–245,728).
	245,721	This chromosome break falls within a repeat ERE2_EH (at coordinates 245,627–245,728).
	245,809	
	245,994	This breakpoint is six nucleotides different from that reported in H_2_O_2_-treated NP69 cells (at coordinate 246,000) [97].

The breakpoints identified within the *AF9* gene were mapped according to the *AF9* sequence retrieved from Ensembl database [EMBL:ENSG00000171843]

**Table 8:** Breakpoints detected within the *AF9* SAR region in BA-treated TWO4 cells

BA-treated TWO4 cells	Breakpoint	Remarks
0.5 mM BA, pH 7.4	245,596	This breakpoint is identical with a breakpoint identified in NP69 cells treated with BA at pH 7.4. This chromosome break falls within the *AF9* region (at coordinates 245,252–245,612) previously reported to be involved in the formation of the *MLL*-*AF9* fusion gene in an ALL patient [GenBank:AM050804]. This breakpoint is three nucleotides different from that reported in cultured normal blood cells treated with VP16 (at coordinate 245,593) [101], five nucleotides different from that detected in H_2_O_2_-treated NP69 cells (at coordinate 245,591) and six nucleotides different from that identified in H_2_O_2_-treated HK1 cells (at coordinate 245,590) [97].
	245,664	This breakpoint is five nucleotides different from that (at coordinate 245,659) identified in NP69 cells treated with H_2_O_2_ [97]. This chromosome break falls within a repeat ERE2_EH (at coordinates 245,627–245,728).
	245,711	This chromosome break falls within a repeat ERE2_EH (at coordinates 245,627–245,728).
	245,769	
	245,803	This breakpoint is identical with that identified in TWO4 cells treated with BA at pH 5.8.
	245,913	
	245,935	
	245,944	
0.5 mM BA, pH 5.8	245,509	This chromosome break falls within the *AF9* region (at coordinates 245,252–245,612) that was previously reported to translocate with the *MLL* gene leading to the formation of the *MLL*-*AF9* fusion gene in an ALL patient [GenBank:AM050804].
	245,577	This chromosome break falls within the *AF9* region (at coordinates 245,252–245,612) that was previously reported to translocate with the *MLL* gene leading to the formation of the *MLL*-*AF9* fusion gene in an ALL patient [GenBank:AM050804].
	245,594	This chromosome break falls within the *AF9* region (at coordinates 245,252–245,612) that was previously reported to translocate with the *MLL* gene leading to the formation of the *MLL*-*AF9* fusion gene in an ALL patient [GenBank:AM050804]. This breakpoint is one nucleotide different from that reported in cultured normal blood cells treated with VP16 (at coordinate 245,593) [101], three nucleotides different from that detected in H_2_O_2_-treated NP69 cells (at coordinate 245,591), and four nucleotides different from that identified in H_2_O_2_-treated HK1 cells (245590) [97].
	245,612	This breakpoint is identical with the breakpoint previously identified in an ALL patient [GenBank:AM050804].
	245,637	This chromosome break falls within a repeat ERE2_EH (at coordinates 245,627–245,728).
	245,729	
	245,753	
	245,803	This breakpoint is identical with a breakpoint identified in TWO4 cells treated with BA at pH 7.4, and seven nucleotides different from that (at coordinate 245,796) reported in NP69 cells treated with H_2_O_2_ [97].
	246,006	This breakpoint is six nucleotides different from that (at coordinate 246,000) reported in NP69 cells treated with H_2_O_2_ [97].
	246,033	
	246,044	
	246,116	This breakpoint is two nucleotides different from a breakpoint (at coordinate 246,114) reported in CEM cells exposed to VP16 [101], and three nucleotides different from that (at coordinate 246,113) identified in NP69 cells treated with H_2_O_2_ [97].
	246,131	

The breakpoints identified within the *AF9* gene were mapped according to the *AF9* sequence retrieved from Ensembl database [EMBL:ENSG00000171843]

**Fig. 6:**
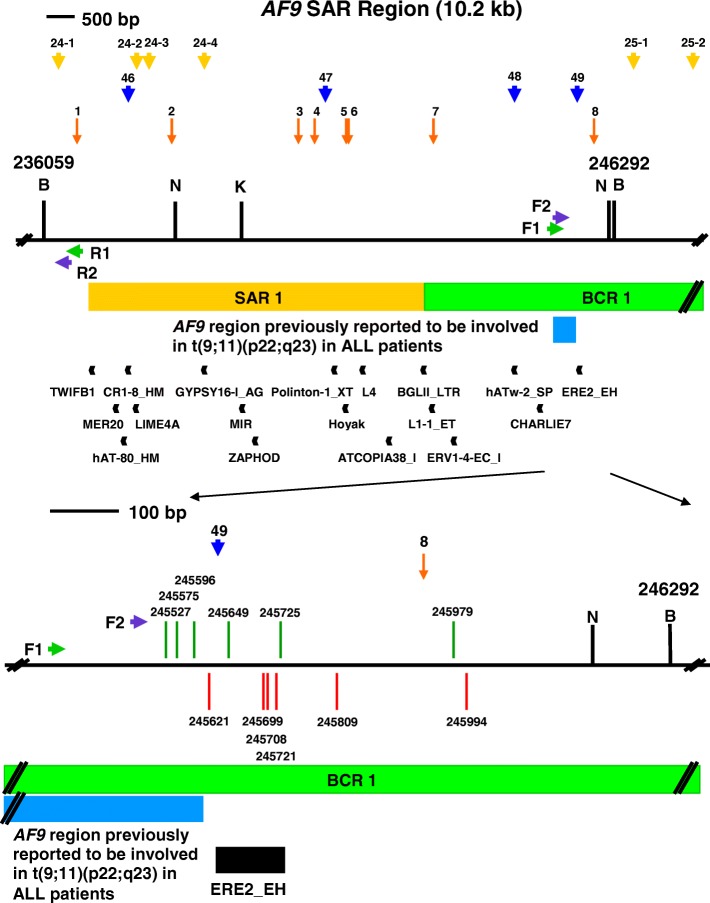
Positions of chromosome breaks within the SAR region in BA-treated NP69 cells. The genomic map of *AF9* SAR region from nucleotide positions 236,059–246,292 is illustrated above [EMBL:ENSG00000171843]. *Bam*H I (B), *Kpn* I (K) and *Nde* I (N) restriction sites are shown. Green arrows represent the primers (R1, AF9 236,451 R and F1, AF9 245,385 F) used in the first round of nested IPCR while the blue arrows represent the primers (R2, AF9 236,211 R and F2, AF9 245,507 F) used in the second round of nested IPCR. Green box represents the previously reported patient BCR which is indicated as BCR1 [88]. Yellow box shows the experimentally determined MAR/SAR which is indicated as SAR1 [88]. Yellow arrows represent the potential MAR/SARs predicted in this study. Blue box represents the *AF9* region (at coordinates 245,252–245,612) previously reported to translocate with the *MLL* gene resulting in the *MLL-AF9* fusion gene identified in an ALL patient [GenBank:AM050804]. Black boxes represent repeat elements. Red and green vertical lines represent the presently detected breakpoints in TWO4 cells treated with BA at neutral and acidic pH, respectively. All the chromosome breaks were mapped within BCR1 in close proximity to SAR1. Three chromosome breaks (at coordinates 245,527, 245,575 and 245,596) fall within the *AF9* region previously reported to be involved in t(9;11)(p22;q23) in the ALL patient. Five chromosome breaks (at coordinates 245,649, 245,699, 245,708, 245,721 and 245,725) fall within a repeat ERE2_EH (at coordinates 245,627–245,728)

**Fig. 7:**
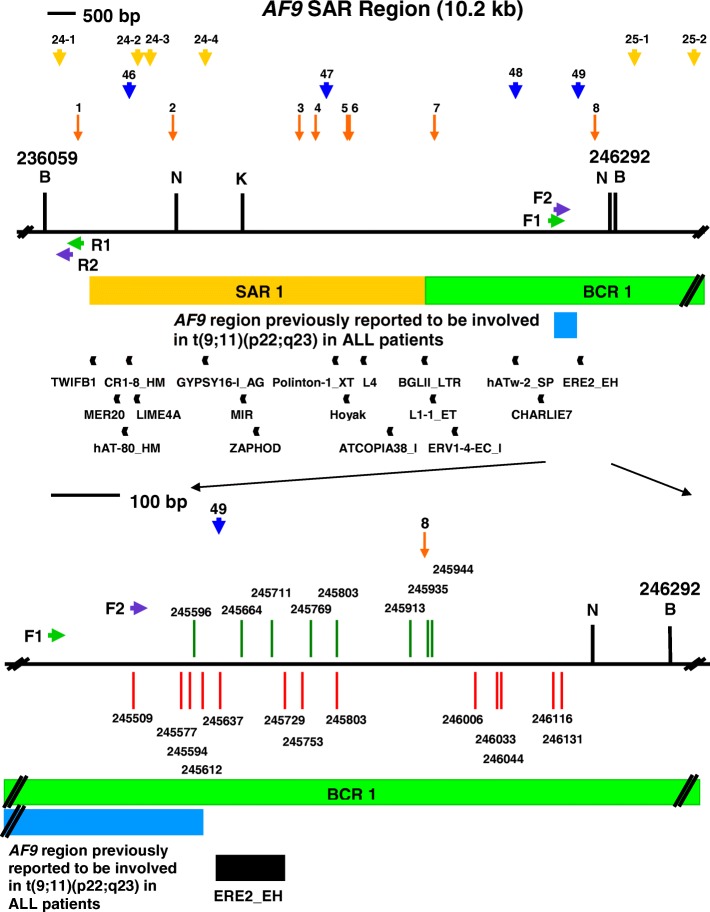
Positions of chromosome breaks within the SAR region in BA-treated TWO4 cells. The genomic map of *AF9* SAR region from nucleotide positions 236,059–246,292 is illustrated above [EMBL:ENSG00000171843]. *Bam*H I (B), *Kpn* I (K) and *Nde* I (N) restriction sites are shown. Green arrows represent the primers (R1, AF9 236,451 R and F1, AF9 245,385 F) used in the first round of nested IPCR while the blue arrows represent the primers (R2, AF9 236,211 R and F2, AF9 245,507 F) used in the second round of nested IPCR. Green box represents the previously reported patient BCR which is indicated as BCR1 [88]. Yellow box shows the experimentally determined MAR/SAR which is indicated as SAR1 [88]. Yellow arrows represent the potential MAR/SARs predicted in this study. Blue box represents the *AF9* region (at coordinates 245,252–245,612) previously reported to translocate with the *MLL* gene resulting in the *MLL-AF9* fusion gene identified in an ALL patient [GenBank:AM050804]. Black boxes represent repeat elements. Red and green vertical lines represent the presently detected breakpoints in TWO4 cells treated with BA at neutral and acidic pH, respectively. All the chromosome breaks were mapped within BCR1 in close proximity to SAR1. Five chromosome breaks (at coordinates 245,509, 245,577, 245,594, 245,596 and 245,612) fall within the *AF9* region previously reported to be involved in t(9;11)(p22;q23) in the ALL patient. One of those breakpoints is identical with that previously identified in the ALL patient (at coordinate 245,612) [GenBank:AM050804]. Three chromosome breaks (at coordinates 245,637, 245,664 and 245,711) fall within a repeat ERE2_EH (at coordinates 245,627–245,728). Two chromosome breaks fall at the same nucleotide position (at coordinate 245,803)

A breakpoint (at coordinate 245,596) was simultaneously mapped in both NP69 and TWO4 cells treated with BA at neutral pH. Another breakpoint (at coordinate 245,803) was simultaneously detected in TWO4 cells treated with BA at both neutral and acidic pH. Three breakpoints (at coordinates 245,594, 245,596 and 246,116) are similar with those reported in cultured normal blood cells (at coordinate 245,593) and CEM cells (at coordinate 246,114) exposed to etoposide (VP16) [101]. A few breakpoints (at coordinates 245,594, 245,596, 245,664, 245,699, 245,708, 245,803, 245,994, 246,006 and 246,116) are similar with those identified in H_2_O_2_-treated NP69 cells (at coordinates 245,591, 245,659, 245,703, 245,796, 246,000 and 246,113) and HK1 cells (at coordinate 245,590) reported in our previous study [97]. In addition, a few chromosome breaks (at coordinates 245,637, 245,649, 245,664, 245,699, 245,708, 245,711, 245,721 and 245,725) fall within a repeat ERE2_EH (at coordinates 245,627–245,728). A map representing the positions of BA-induced chromosome breaks in NP69 and TWO4 cells within the *AF9* SAR region is illustrated in Figs. 6 and 7, respectively.

**Fig. 8:**
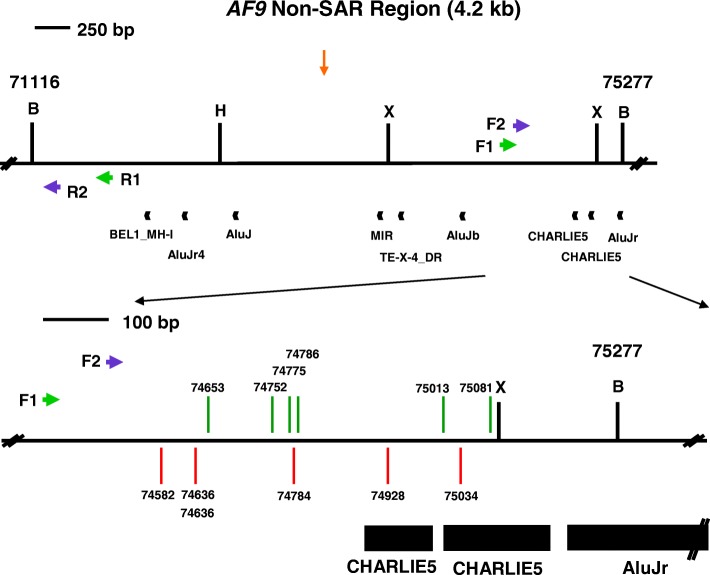
Positions of chromosome breaks within the non-SAR region in BA-treated NP69 cells. The genomic map of *AF9* non-SAR region from nucleotide positions 71,116–75,277 is illustrated above [EMBL: ENSG00000171843]. *Bam*H I (B), *Hin*d III (H) and *Xba* I (X) restriction sites are shown. Green arrows represent the primers (R1, AF9 71,653 R and F1, AF9 74,399 F) used in the first round of nested IPCR while blue arrows represent the primers (R2, AF9 71,282 R and F2, AF9 74,494 F) used in the second round of nested IPCR. Black boxes represent the repeat elements. Red and green vertical lines represent the presently identified breakpoints in NP69 cells upon BA treatment at neutral and acidic pH, respectively. One chromosome break (at coordinate 74,928) falls within the first repeat CHARLIE5 (at coordinates 74,895–74,998). Three chromosome breaks (at coordinates 75,013, 75,034 and 75,081) fall within the second repeat CHARLIE5 (at coordinates 75,006–75,169). Two chromosome breaks fall at the same nucleotide position (at coordinate 74,636)

**Fig. 9:**
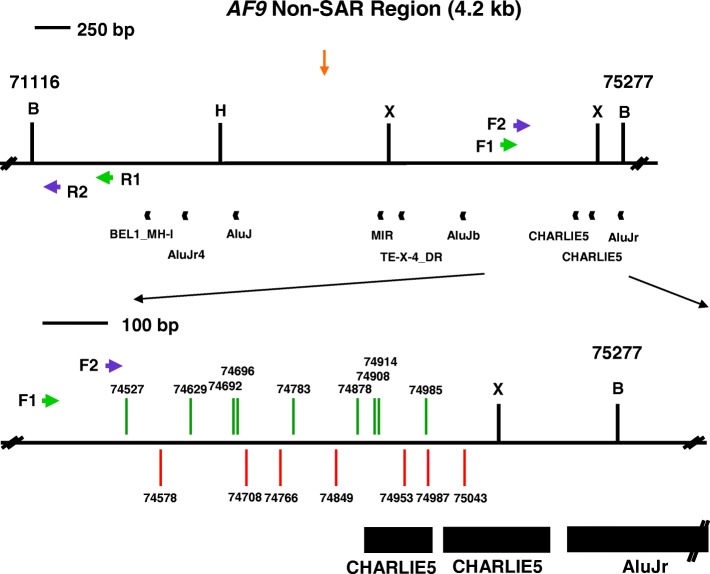
Positions of chromosome breaks within the non-SAR region in BA-treated TWO4 cells. The genomic map of *AF9* non-SAR region from nucleotide positions 71,116–75,277 is illustrated above [EMBL: ENSG00000171843]. *Bam*H I (B), *Hin*d III (H) and *Xba* I (X) restriction sites are shown. Green arrows represent the primers (R1, AF9 71,653 R and F1, AF9 74,399 F) used in the first round of nested IPCR while blue arrows represent the primers (R2, AF9 71,282 R and F2, AF9 74,494 F) used in the second round of nested IPCR. Black boxes represent the repeat elements. Red and green vertical lines represent the presently detected breakpoints in TWO4 cells upon BA treatment at neutral and acidic pH, respectively. Five chromosome breaks (at coordinates 74,908, 74,914, 74,953, 74,985 and 74,987) fall within the first repeat CHARLIE5 (at coordinates 74,895–74,998). One chromosome break (at coordinate 75,043) falls within the second repeat CHARLIE5 (at coordinates 75,006–75,169)

Tables 9 and 10 show the breakpoints identified within the *AF9* non-SAR region in BA-treated NP69 and TWO4 cells, respectively. One breakpoint was simultaneously detected in two different IPCR replicates which were from two different sets of IPCR derived from NP69 treated with BA at acidic pH. Six chromosome breaks (at coordinates 74,908, 74,914, 74,928, 74,953 and 74,987) fall within the first repeat CHARLIE5 (at coordinates 74,895–74,998). Four chromosome breaks (at coordinates 75,013, 75,034, 75,043 and 75,081) fall within the second repeat CHARLIE5 (at coordinates 75,006–75,169). The genomic maps representing the positions of chromosome breaks within the *AF9* non-SAR region detected in NP69 and TWO4 cells are elucidated in Figs. 8 and 9, respectively.

**Table 9:** Breakpoints detected within the *AF9* non-SAR region in BA-treated NP69 cells

BA-treated NP69 cells	Breakpoint	Remarks
0.5 mM BA, pH 7.4	74,653	
	74,752	
	74,775	
	74,786	
	75,013	This chromosome break falls within a repeat CHARLIE5 (at coordinates 75,006–75,169).
	75,081	This chromosome break falls within a repeat CHARLIE5 (at coordinates 75,006–75,169).
0.5 mM BA, pH 5.8	74,582	
	74,636	Identical breakpoints detected in different IPCR replicates which were from two different sets of IPCR.
	74,784	
	74,928	This chromosome break falls within a repeat CHARLIE5 (at coordinates 74,895–74,998).
	75,034	This chromosome break falls within a repeat CHARLIE5 (at coordinates 75,006–75,169).

The nucleotide positions of the chromosome breaks detected within the *AF9* gene were mapped according to the *AF9* sequence accessed from Ensembl database [EMBL:ENSG00000171843]

**Table 10:** Breakpoints detected within the *AF9* non-SAR region in BA-treated TWO4 cells

BA-treated TWO4 cells	Breakpoint	Remarks
0.5 mM BA, pH 7.4	74,527	
	74,629	
	74,692	
	74,696	
	74,783	
	74,878	
	74,908	This chromosome break falls within a repeat CHARLIE5 (at coordinates 74,895–74,998).
	74,914	This chromosome break falls within a repeat CHARLIE5 (at coordinates 74,895–74,998).
	74,985	This chromosome break falls within a repeat CHARLIE5 (at coordinates 74,895–74,998).
0.5 mM BA, pH 5.8	74,578	
	74,708	
	74,766	
	74,849	
	74,953	This chromosome break falls within a repeat CHARLIE5 (at coordinates 74,895–74,998).
	74,987	This chromosome break falls within a repeat CHARLIE5 (at coordinates 74,895–74,998).
	75,043	This chromosome break falls within a repeat CHARLIE5 (at coordinates 75,006–75,169).

The nucleotide positions of the chromosome breaks detected within the *AF9* gene were mapped according to the *AF9* sequence accessed from Ensembl database [EMBL:ENSG00000171843]

## Discussion

Lately, the association between chronic inflammation of sinonasal tract and NPC has increasingly received much attention [15]. One of the major risk factors for the development of CRS is GORD [42, 43, 102]. It has been demonstrated that gastric duodenal refluxate can reach the larynx, pharynx, oral cavity, nasopharynx, nose, sinus, eustachian tube and middle ear. Repeated exposure to gastric duodenal content may result in localised inflammation of these regions [18, 20, 43, 103–106]. More recently, BA has been shown to cause cell injury and inflammation in the airway epithelium. Treatment of the immortalised human bronchial epithelial cells (BEAS-2B) with BA resulted in increased activity of proinflammatory cytokines (interleukin-8 and interleukin-6) [107]. The airways do not have intrinsic protective mechanisms as found in the oesophagus. Therefore, the tissues of airways are more vulnerable to acid-peptic injury as compared with the oesophagus. Due to this reason, it is conceivable that when the tissues of airways are repeatedly exposed to the gastric duodenal refluxate, the genotoxicity and mutagenicity of gastric duodenal content may also contribute to carcinogenesis in the airways [108].

By using flow cytometric analyses of phosphatidylserine (PS) externalisation and mitochondrial membrane potential (MMP) disruption, we have previously demonstrated that BA induced apoptosis in normal nasopharyngeal epithelial cells (NP69) and NPC cells (TWO4) [75]. We further demonstrated that BA-induced apoptosis triggered oxidative stress and caspase activity. These events, in turn, resulted in cleavages within the *AF9* BCR. These cleavages were abolished by the caspase inhibitor, suggesting that these cleavages were mediated CAD. Our findings suggested that one of the potential mechanisms contributing to chromosome rearrangement in NPC could be BA-induced apoptosis, where CAD may be involved [75]. In the present report, we intended to investigate the relation between the positions of BA-induced chromosomal cleavages and the chromatin structure.

It has been known that the BCRs of the *AF9* and *MLL* genes share similar structural elements. These structural elements include MAR/SAR, topo II cleavage site and DNase I hypersensitive site. The similarity in the structural features in the BCRs of the *AF9* and *MLL* genes has been suggested to serve as recombination hot spots leading to the formation of the *MLL*-*AF9* fusion gene in leukaemogenesis [88]. MAR/SARs are DNA sequences that are responsible for chromosomal loop attachment [109]. Topo II cleavage site and DNase I hypersensitive site frequently co-localise with MAR/SAR [109–111]. Therefore, we attempted to study the role of MAR/SAR in determining the positions of chromosomal cleavages in BA-induced apoptosis.

The targeted gene in this study was the *AF9* gene located on the short arm of chromosome 9 at position 9p22, a common deletion region in NPC. The *AF9* gene frequently translocates with the *MLL* gene at 11q23 resulting in the reciprocal translocation t(9;11)(p22;q23) in leukaemia [88]. The fusion of these two genes has been found to occur predominantly among the patients with de novo acute myelogenous leukaemia (AML). The *MLL*-*AF9* fusion gene was less commonly observed in patients with acute lymphocytic leukaemia (ALL), with myelodysplastic syndrome (MDS) and with therapy-related AML (t-AML) [88, 112].

In the present study, possible MAR/SAR sites in the *AF9* gene were predicted by using MRS. MRS is a bipartite sequence element which has been strongly associated with MAR/SARs. MRS is composed of two individual sequence elements which are found together within a distance of approximately 200 bp. However, when the DNA is wrapped around the histones, these two sequence elements exist at a location near the dyad axis of the nucleosome. Hence, they are found parallel to each other in MAR/SAR when the nucleosomes are positioned. This allows them to generate a protein binding site in MAR/SAR. van Drunen and co-workers have analysed more than 300 kb of DNA sequences from several eukaryotic organisms by using MRS. Their studies reported that all the MRS predictions were associated with the experimentally determined MAR/SARs [87]. MRS has been widely used in other studies and proved to be reliable [113–115].

The MRS predictions obtained in the present study were compared with the location of experimentally determined MAR/SARs reported in the previous studies [88, 89]. Strissel and co-workers have analysed exons 4 to 10 of the *AF9* gene for MAR/SAR. In this region of 61 kb in length, two biochemically extracted MAR/SARs have been reported. These two MAR/SARs were indicated as SAR1 and SAR2. SAR1 is a 6.2 kb MAR/SAR found in intron 4. SAR2 is a 4.6 kb MAR/SAR spans through parts of introns 5 to 7 [88]. To the best of our knowledge, there is no previous report on the experimentally determined MAR/SAR for the *AF9* region from exon 1 to intron 3. Four MRS predictions (MAR/SARs 24–1 to 24–4 in Fig. 1) are associated with SAR1. One of these four MRSs was located in a region < 1 kb centromeric to SAR1 (MAR/SAR 24–1 in Fig. 1), whereas the other three MRSs were found within SAR1 (MAR/SARs 24–2 to 24–4 in Fig. 1). One MRS prediction (MAR 27 in Fig. 1) correlates with SAR2. This MRS was found in a region < 1.5 kb telomeric to SAR2. Interestingly, all the MRS-predicted MAR/SARs were found in the introns of the *AF9* gene. These results are consistent with those of the other studies which found that MAR/SARs occur more frequently in introns than in exons. This has been previously confirmed by both the experimental extraction [116, 117] and computational prediction [91].

Based on the in silico prediction and the previous studies which reported the experimentally determined MAR/SARs [88], the SAR and non-SAR regions were determined (Fig. 1). A study by van Drunen and colleagues (1999) showed that they never found a MRS which did not correlate with an experimentally verified MAR/SAR. However, their studies also revealed that not all biochemically defined MAR/SARs contain a MRS. Their findings suggested that there is at least a distinct type of MAR/SAR which does not contain a MRS [87]. Thus, in order to investigate if the region which was considered as a non-SAR region contains another type of MAR/SAR which was not predicted by MRS, we further analysed the *AF9* sequence by predicting the presence of MAR/SAR using MAR-Finder and SMARTest.

The MAR/SAR analysis rules utilised in these two programs are different from the criteria used in the MAR/SAR prediction by MRS. MAR-Finder utilises statistical inference to predict the occurrence of MAR/SARs. MAR-Finder was developed by using the formulation of a set of biological rules based on the correlation of MAR/SAR with various DNA sequence motifs. These motifs include the TG-rich sequences, origin of replication (ORI), kinked DNA, curved DNA, AT-rich sequences and topo II sites. MAR-Finder has been shown to successfully identify MAR/SAR sites which correlate with those experimentally verified in the human beta-globin gene, PRM1-PRM2-TNP2 domains and human apolipoprotein B locus [92]. By contrast, SMARTest predicts MAR/SARs based on a density analysis of MAR/SAR-associated features described by a weight matrix library [91]. This MAR/SAR matrix library was mainly derived from the following MAR/SAR-associated patterns. Firstly, MAR/SARs have a minimum sequence length of 200–300 bp [118]. Secondly, MAR/SAR sequences are AT-rich [117]. Thirdly, MAR/SARs are associated with a few motifs. These motifs include ATTA, ATTTA, AATATT, ATATTT and AATATATTT [85, 118–121].

In order to evaluate the capability of SMARTest, Frisch and co-workers analysed six genomic sequences (three human sequences and three plant sequences) with a total of 310 kb in length by using SMARTest. These six genomic sequences contain a total of 37 experimentally determined MAR/SARs. Their studies showed that 19 of 28 SMARTest predictions were true positives (specificity = 68%). These 19 true positives only correlate with 14 of 37 biochemically extracted MAR/SARs in these genomic sequences (sensitivity = 38%), as some of the MAR/SARs contain more than one SMARTest predictions. For comparison, the authors analysed the same six sequences by using MAR-Finder. Twenty of 25 MAR-Finder predictions were true positives (specificity = 80%). These 20 true positives only correlate with 12 of 37 biochemically extracted MAR/SARs in these sequences, as some of the MAR/SARs contain more than one MAR-Finder predictions (sensitivity = 32%) [91]. Given that the MAR/SAR matrix library utilised by SMARTest was derived from AT-rich MAR/SAR, other MAR/SAR classes distinct from AT-rich class were not predicted by SMARTest. However, these MAR/SAR classes distinct from AT-rich class were found by MAR-Finder. Frisch and co-workers further found that some of the experimentally determined MAR/SARs which were not identified by MAR-Finder were detected by SMARTest. Hence, SMARTest and MAR-Finder were suggested to mutually complete each other in MAR/SAR prediction [91].

Due to a lack of report on the experimentally determined MAR/SARs for the *AF9* region from exon 1 to intron 3 (approximately 220 kb in length), the sensitivity and specificity of MRS, SMARTest and MAR-Finder were unable to be compared in this study. Nevertheless, the comparison of accuracy among these prediction tools was not the aim of this study. The main purpose of using these different MAR/SAR prediction tools was to predict the MAR/SARs of different classes. By using MRS, SMARTest and MAR-Finder for MAR/SAR prediction, our findings suggested that the non-SAR region does not contain any MAR/SAR (Fig. 1).

Given that chromosomal cleavage is an initial event in both apoptosis and chromosome rearrangements, we employed nested IPCR to identify the chromosome breaks mediated by BA-induced apoptosis. Our findings showed that, for the SAR region, the cleavage frequencies in BA-treated cells were significantly higher than those in the untreated control. On the contrary, for the non-SAR region, there was no significant difference in the cleavage frequencies between the BA-treated cells and untreated control cells. These observations were true for both NP69 and TWO cell lines. However, in both untreated NP69 and TWO4 cells, the cleavage frequencies of the non-SAR region were significantly higher than those of the SAR region. By using CENSOR program, we found that the overall content of repeat elements in the non-SAR region is 3.0-fold higher (41.37% vs 13.81%) than that in the SAR region. Considering that no significant difference in the cleavage frequencies of the non-SAR region was found between the untreated and BA-treated cells, it seems not unlikely that the chromosome breaks in the non-SAR region were not mediated by BA-induced apoptosis. Rather, the chromosome breaks detected in this region were mostly spontaneous breaks due to DNA fragility contributed by these repeat elements. It is likely that repeat elements make the chromosome to be more prone to cleavage. These results are consistent with those of the other studies which reported a high proportion of repeat elements in common fragile sites, including FRA3B, FRA7G, FRA7H, FRA16D and FRAXB. These repeat elements include interspersed repeat elements, long terminal repeats (LTR), transposable elements, Mirs, L1 elements, L2 elements and *Alu* elements [122, 123]. Therefore, we concluded that MAR/SAR may play an essential role in mediating the gene cleavages in BA-induced apoptosis in NP69 and TWO4 cells at both neutral and acidic pH.

Knowing that topo II was involved in mediating illegitimate recombination [124, 125], we further analysed the SAR and non-SAR regions for topo II consensus sites. The topo II consensus sites were predicted by using an 18 bp consensus sequence [98, 99]. Our findings showed that the proportion of topo II sites in the SAR region was approximately 3-fold higher than that in the non-SAR region (1.41% vs 0.43%). These results seemed to reaffirm the findings of those studies which unravelled that MAR/SARs are the dominant sites for topo II binding and cleavage [109, 126].

In an in vitro system, topo II has been demonstrated to play a critical role in mediating DNA cleavages at acidic pH. Topo II has also been shown to be involved in mediating mutation and cytotoxicity induced by acidic pH in tissue culture models. These findings suggested that topo II-mediated DNA damage may lead to the development of cancers associated with gastro-oesophageal acid reflux [127]. In addition, previous studies have demonstrated that topo II is responsible for chromosomal loops excision in the early stage of apoptosis induced by oxidative stress. This initial event was followed by activation of nucleases leading to degradation of chromosomal DNA into nucleosomal DNA [128].

Our previous study has shown that BA and/or acidic pH induced apoptosis via oxidative stress in nasopharyngeal epithelial cells. In BA-induced apoptosis, we demonstrated that DNA cleavages within the SAR region occurred in a caspase-3-dependent manner, suggesting that CAD is responsible for these DNA cleavages [75]. Besides, we have also previously demonstrated that CAD cleaves the DNA preferentially at MAR/SAR sites during oxidative stress [97]. It has been observed that CAD was closely associated with the nuclear matrix of apoptotic cells [84]. It is conceivable that when CAD binds to the nuclear matrix during apoptosis, CAD preferentially cleaves the DNA at MAR/SAR sequences. It is possible that in BA and/or acidic pH-induced apoptosis, which involves oxidative stress, both topo II and CAD do play a role in mediating the DNA cleavages. The former may take part in mediating the cleavage of loop-sized DNA into HMW fragments whereas the latter may involve in mediating the degradation of chromosomal DNA into nucleosomal DNA. Therefore, our current findings which revealed that BA-induced apoptosis resulted in DNA cleavages within the SAR region may be explained by the close relation among topo II, CAD and MAR/SAR.

Sequencing of IPCR bands detected in the SAR region showed the positions of chromosome breaks within the *AF9* BCR1 mediated by BA-induced apoptosis. The *AF9* BCR1 is bordered by SAR1 and SAR2 [88, 89]. It is noteworthy that the positions of the chromosome breaks identified in the present study were highly similar to those previously reported in leukaemia patients. A few chromosome breaks were mapped within the *AF9* region that was previously reported to translocate with the *MLL* gene in an ALL patient. This reciprocal translocation t(9;11)(p22;q23) resulted in the formation of *MLL*-*AF9* fusion gene in the ALL patient [GenBank:AM050804]. Additionally, we identified a breakpoint which is identical with that identified in the ALL patient [GenBank:AM050804].

## Conclusions

In summary, our current results reaffirm our previous findings that BA-induced apoptosis may cause chromosomal breakages in nasopharyngeal epithelial cells. In addition, our findings further implicate that MAR/SAR, which has a close association with topo II and CAD, plays a critical role in determining the positions of BA-induced chromosomal breakages. The positions of these BA-induced chromosome breaks shared high similarity with those identified in patients with leukaemia. Given that chromosomal breakage is an initial event of chromosome rearrangement and that cells may survive apoptosis upon compromised DNA repair, repeated exposure of nasopharyngeal epithelial cells to acid refluxate may contribute to genomic instability. The elevated mutation rate may in turn lead to the development of NPC. In order to clarify the relation between GOR and NPC, there are a few questions remain to be answered by further investigations: (i) Whether GOR may directly contribute to the pathogenesis of NPC through the cytotoxicity and genotoxicity driven by acid refluxate? (ii) Whether GOR may indirectly contribute to the pathogenesis of NPC through chronic inflammation of sinonasal tract (such as CRS) that has been recognised as a precursor of NPC? (iii) Whether both chronic inflammation of sinonasal tract and NPC share a similar underlying mechanism contributed by GOR? Nevertheless, our findings have unfolded the potential role of refluxed gastro-duodenal contents in contributing to NPC chromosome rearrangements.

## Additional files

Additional file 1: Flow chart depicting the simplified DNA manipulation steps in preparation for nested IPCR. (PDF 46 kb)

Additional file 2: Description of exons and introns in the *AF9* gene. (PDF 64 kb)

## Abbreviations

*ABL*: Abelson murine leukaemia viral oncogene homolog 1
ALL: Acute lymphoblastic leukaemia
AML: Acute myelogenous leukaemia
BA: Bile acid
CAD: Caspase-activated DNase
CD: Cluster of differentiation
CRS: Chronic rhinosinusitis
EBV: Epstein-Barr virus
EMT: Epithelial-mesenchymal transition
EOR: Extraoesophageal reflux
GOR: Gastro-oesophageal reflux
GORD: Gastro-oesophageal reflux disease
H_2_O_2_: Hydrogen peroxide
HMW: High-molecular-weight
IFN: Interferon
IL: Interleukin
IPCR: Inverse-PCR
MAR/SAR: Matrix association region/scaffold attachment region
MDS: Myelodysplastic syndrome
*MLL*: Mixed lineage leukaemia
MMP: Mitochondrial membrane potential
MRS: MAR/SAR recognition signature
NF-kappa B: Nuclear factor-kappa B
NPC: Nasopharyngeal carcinoma
ORI: Origin of replication
PS: Phosphatidylserine
ROS: Reactive oxygen species
SD: Standard deviation
SDS: Sodium dodecyl sulphate
t-AML: Therapy-related AML
TNF-alpha: Tumour necrosis factor-alpha
topo II: Topoisomerase II
VP16: Etoposide
