# Supplementary material for: Matrix association region/scaffold attachment region: the crucial player in defining the positions of chromosome breaks mediated by bile acid-induced apoptosis in nasopharyngeal epithelial cells
Source: BMC Med Genomics. 2019 Jan 15;12:9. doi: 10.1186/s12920-018-0465-4 (PMC6334432; doi:10.1186/s12920-018-0465-4)
Supplement: Supplementary file 1 — Flow chart depicting the simplified DNA manipulation steps in preparation for nested IPCR. (PDF 46 kb) [file 12920_2018_465_MOESM1_ESM.pdf]

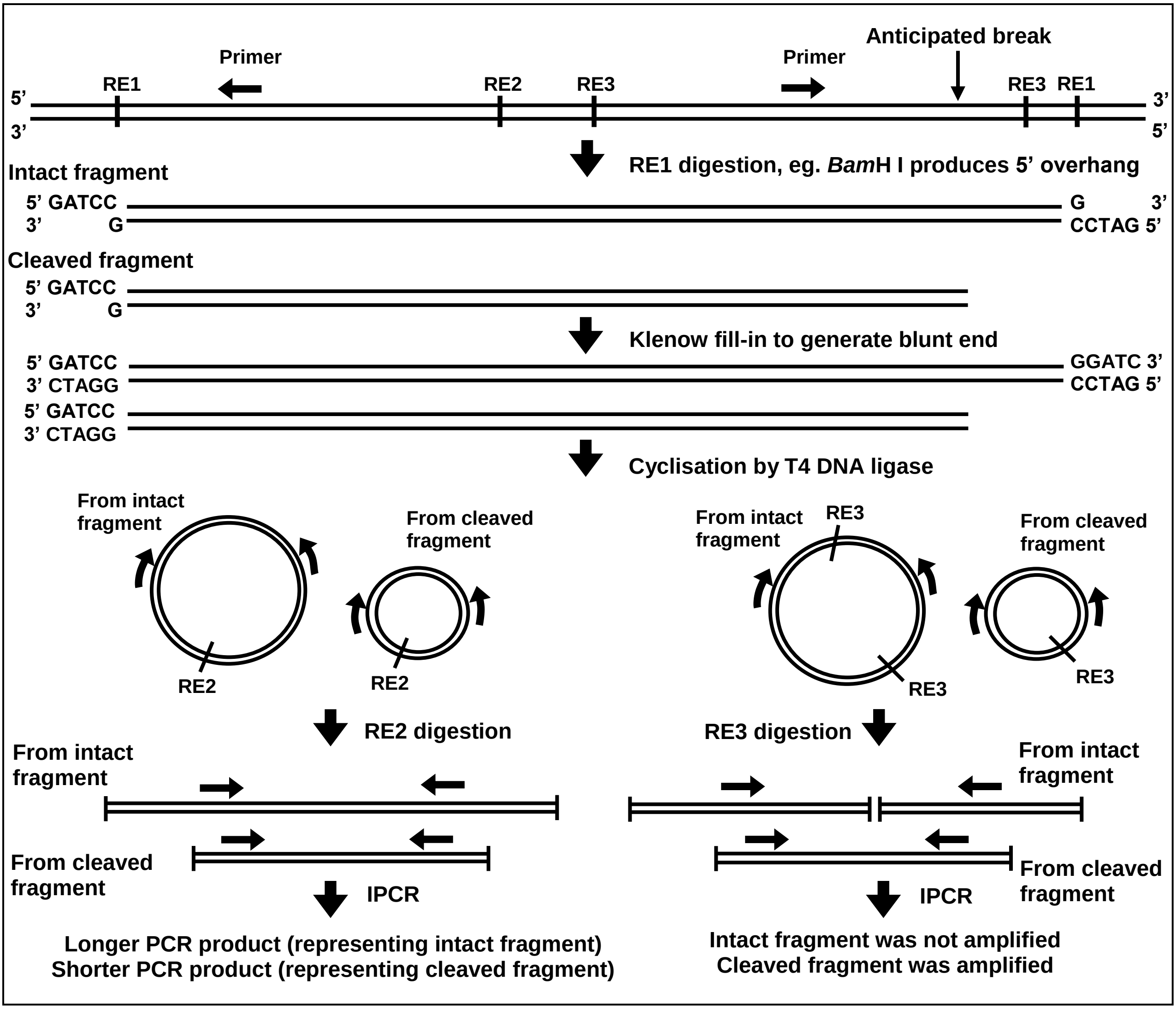

Additional file 1

DNA manipulation steps in preparation for nested IPCR. The extracted genomic DNA was modified through restriction enzyme digestion, Klenow fill-in and cyclisation. Nested IPCR was performed after these manipulation steps [89].
