# Supplementary material for: Matrix association region/scaffold attachment region: the crucial player in defining the positions of chromosome breaks mediated by bile acid-induced apoptosis in nasopharyngeal epithelial cells
Source: BMC Med Genomics. 2019 Jan 15;12:9. doi: 10.1186/s12920-018-0465-4 (PMC6334432; doi:10.1186/s12920-018-0465-4)
Supplement: Supplementary file 2 — Description of exons and introns in the AF9 gene. (PDF 64 kb) [file 12920_2018_465_MOESM2_ESM.pdf]

Additional file 2

Description of exons and introns in the *AF9* gene

Exon/Intron	*Nucleotide position*	*Length (bp)*
5' upstream sequence	1-600	600
Exon 1	601-899	299
Intron 1	900-2309	1410
Exon 2	2310-2490	181
Intron 2	2491-166358	163868
Exon 3a	166359-166441	83
Intron 3a	166442-174878	8437
Exon 3b	174879-175022	144
Intron 3b	175023-208719	33697
Exon 4	208720-209424	705
Intron 4	209425-257400	47976
Exon 5	257401-257476	76
Intron 5	257477-259539	2063
Exon 6	259540-259669	130
Intron 6	259670-262303	2634
Exon 7	262304-262403	100
Intron 7	262404-268265	5862
Exon 8	268266-268337	72
Intron 8	268338-269548	1211
Exon 9	269549-269620	72
Intron 9	269621-276570	6950
Exon 10	276571-281480	4910
3' downstream sequence	281481-282080	600

The *AF9* gene located at 9p22. It is 280880 bp in length [EMBL:ENSG00000171843].
